# Supplementary material for: Drying parameters and aging modulate protective properties of vitrified trehalose
Source: bioRxiv. 2026 Jan 17:2026.01.16.700019. Preprint. [Version 1] doi: 10.64898/2026.01.16.700019 (PMC12871358; doi:10.64898/2026.01.16.700019)
Supplement: 1 [file NIHPP2026.01.16.700019V1-supplement-1.pdf]

bioRxiv preprint doi: https://doi.org/10.64898/2026.01.16.700019; this version posted January 17, 2026. The copyright holder for this preprint (which was not certified by peer review) is the author/funder, who has granted bioRxiv a license to display the preprint in perpetuity. It is made available under aCC-BY 4.0 International license.

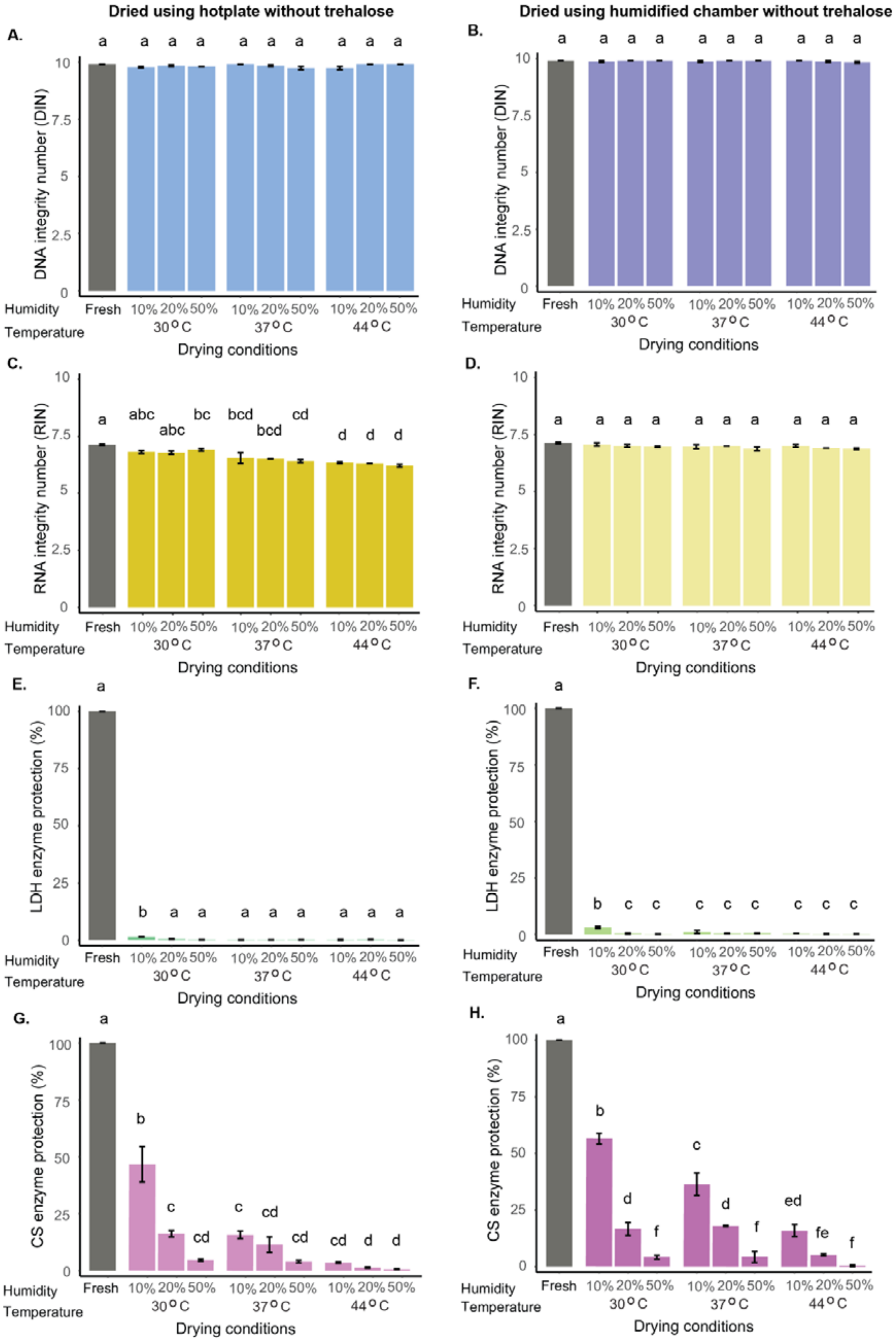

bioRxiv preprint doi: https://doi.org/10.64898/2026.01.16.700019; this version posted January 17, 2026. The copyright holder for this preprint (which was not certified by peer review) is the author/funder, who has granted bioRxiv a license to display the preprint in perpetuity. It is made available under aCC-BY 4.0 International license.

Supplementary Figure S1. Differential stability of nucleic acids and proteins under various drying conditions in the absence of trehalose.
DNA (A,B), RNA (C,D), lactate dehydrogenase (LDH; E,F), and citrate synthase (CS; G,H) samples were dried using two methods-hotplate (A,C,E,G) and humidified chamber (B,D,F,H)-across nine temperature-humidity combinations, all in the absence of 300 mM trehalose. DNA and RNA integrity were assessed using DNA Integrity Number (DIN) and RNA Integrity Number (RIN), respectively, while enzyme activity retention was used to evaluate protein function. Different letters indicate statistically significant differences among drying conditions within each panel, as determined by one-way ANOVA followed by Tukey's post hoc test (α = 0.05). Data represent mean ± SE from three independent replicates per condition.

bioRxiv preprint doi: https://doi.org/10.64898/2026.01.16.700019; this version posted January 17, 2026. The copyright holder for this preprint (which was not certified by peer review) is the author/funder, who has granted bioRxiv a license to display the preprint in perpetuity. It is made available under aCC-BY 4.0 International license.

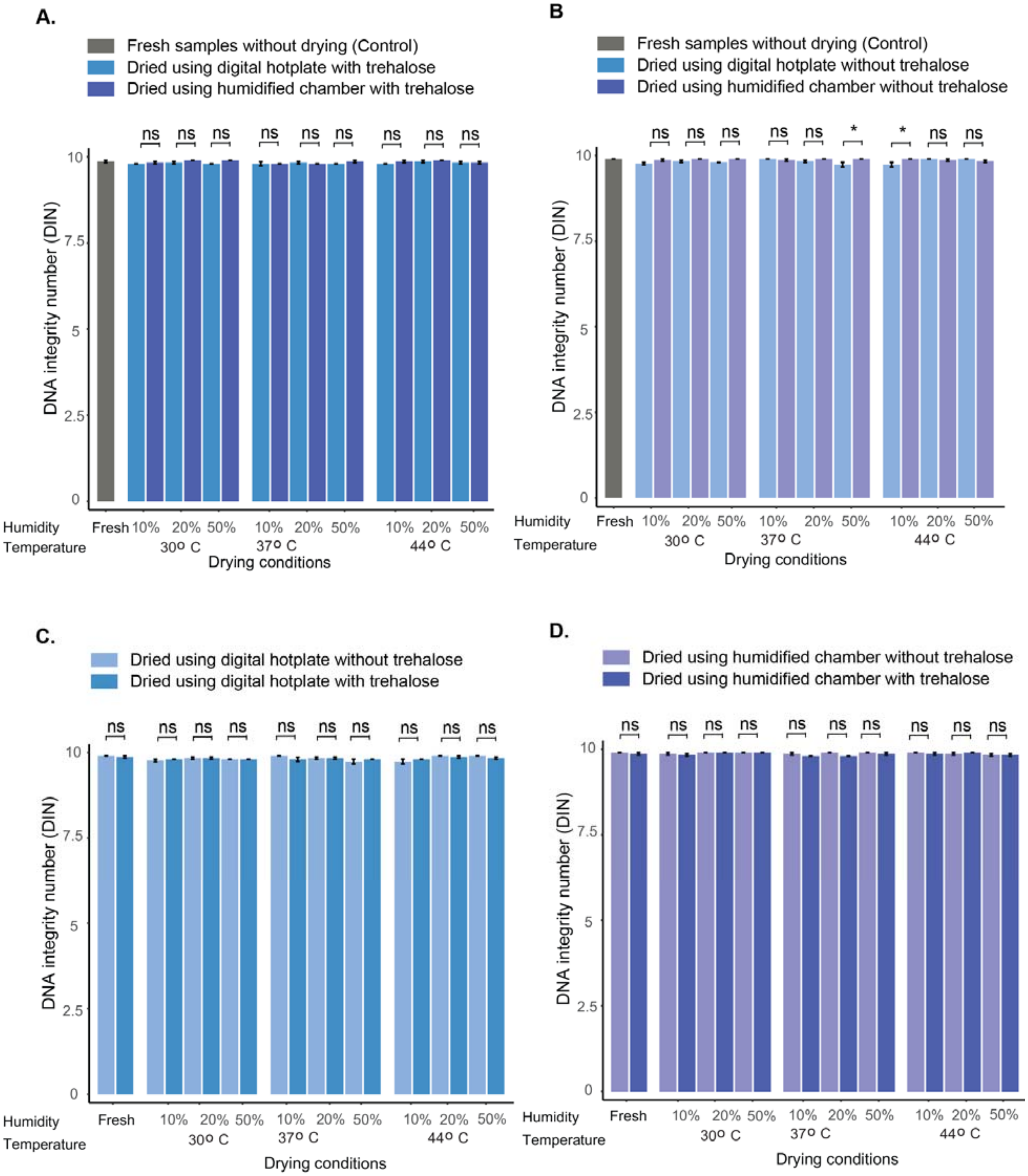

Supplementary Figure S2. Effect of drying method and trehalose on DNA integrity. Comparison of drying methods with trehalose (A). Comparison of drying methods without trehalose (B). Hotplate drying with and without trehalose (C). Humidified chamber drying with and without trehalose (D). DNA integrity was assessed using the DNA Integrity Number (DIN). Statistical comparisons were made only between treatments within the same drying condition, as determined by one-way ANOVA followed by Tukey's post hoc test (α = 0.05). Data represent mean ± SE from three independent replicates per condition.

bioRxiv preprint doi: https://doi.org/10.64898/2026.01.16.700019; this version posted January 17, 2026. The copyright holder for this preprint (which was not certified by peer review) is the author/funder, who has granted bioRxiv a license to display the preprint in perpetuity. It is made available under aCC-BY 4.0 International license.

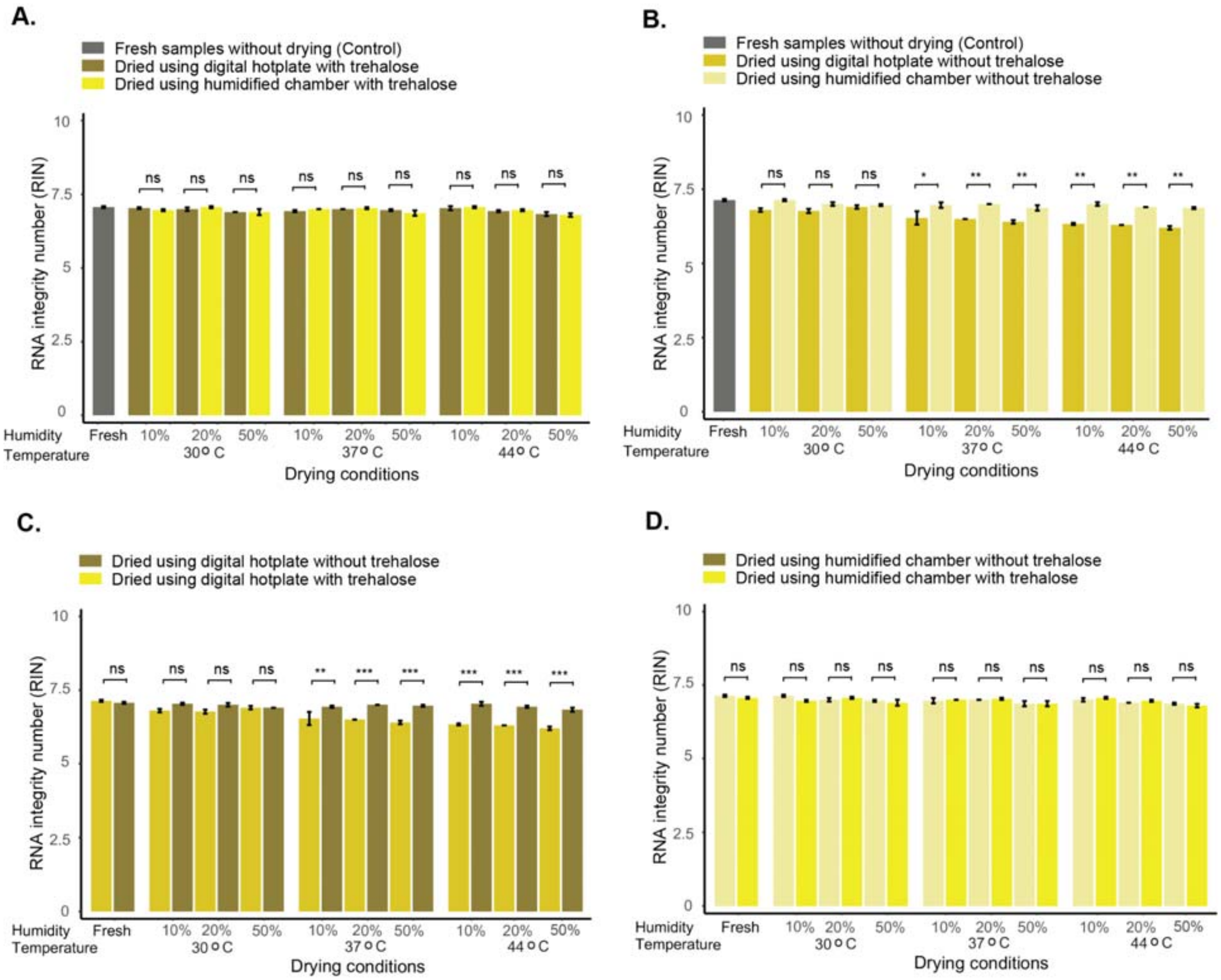

Supplementary Figure S3. Effect of drying method and trehalose on RNA integrity. Comparison of drying methods with trehalose (A). Comparison of drying methods without trehalose (B). Hotplate drying with and without trehalose (C). Humidified chamber drying with and without trehalose (D). RNA integrity was assessed using the RNA Integrity Number (RIN). Statistical comparisons were made only between treatments within the same drying condition, as determined by one-way ANOVA followed by Tukey's post hoc test (α = 0.05). Data represent mean ± SE from three independent replicates per condition.

bioRxiv preprint doi: https://doi.org/10.64898/2026.01.16.700019; this version posted January 17, 2026. The copyright holder for this preprint (which was not certified by peer review) is the author/funder, who has granted bioRxiv a license to display the preprint in perpetuity. It is made available under aCC-BY 4.0 International license.

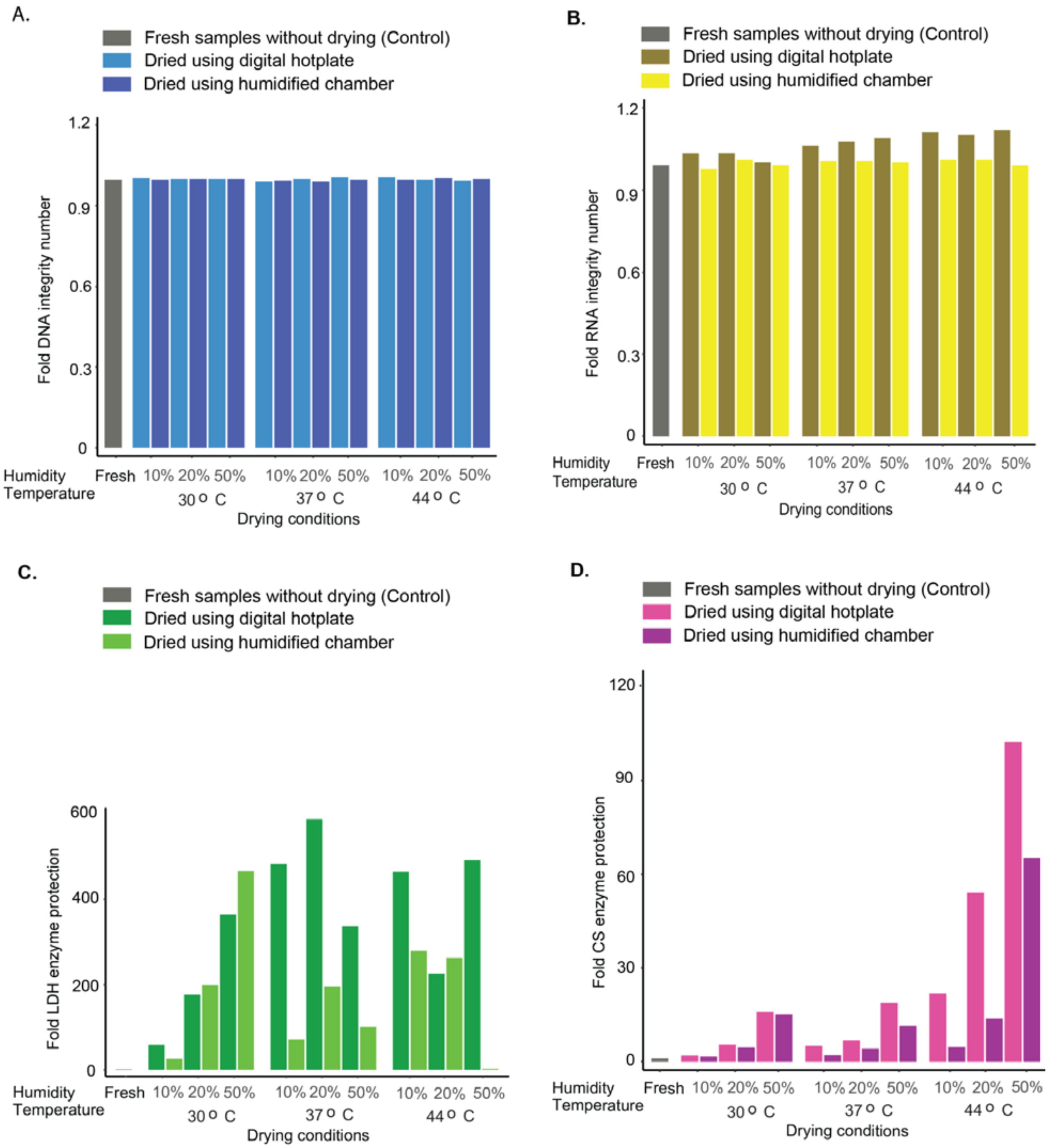

Supplementary Figure S4. Fold protection of nucleic acids and proteins under different drying conditions.

Fold protection of DNA (A), RNA (B), lactate dehydrogenase (LDH; C), and citrate synthase (CS; D) in both drying methods: hotplate and humidified chamber. Fold protection was calculated as the ratio of average biomolecular integrity/activity in samples dried with trehalose to that in samples dried without.

bioRxiv preprint doi: https://doi.org/10.64898/2026.01.16.700019; this version posted January 17, 2026. The copyright holder for this preprint (which was not certified by peer review) is the author/funder, who has granted bioRxiv a license to display the preprint in perpetuity. It is made available under aCC-BY 4.0 International license.

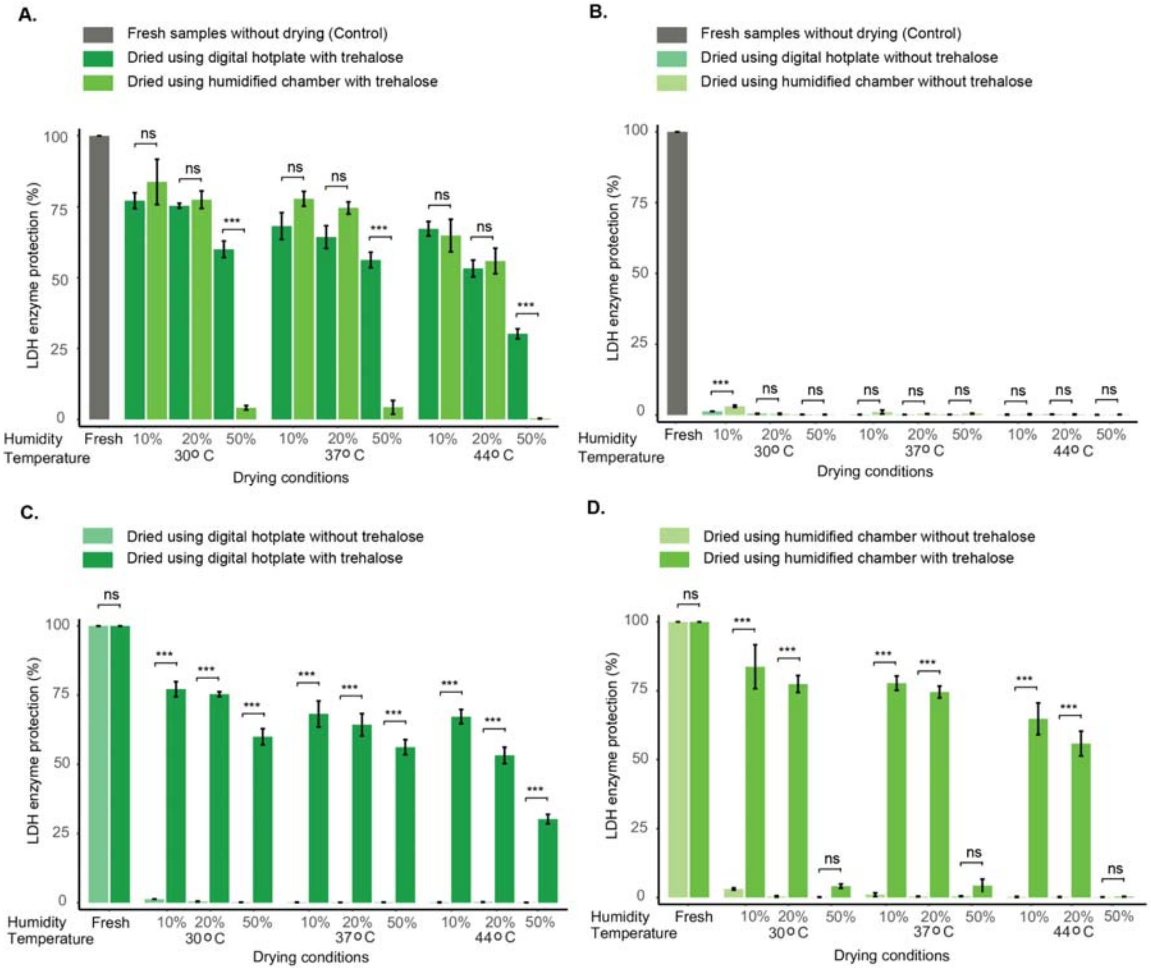

Supplementary Figure S5. Effect of drying method and trehalose on LDH activity. Comparison of drying methods with trehalose (A). Comparison of drying methods without trehalose (B). Hotplate drying with and without trehalose (C). Humidified chamber drying with and without trehalose (D). Lactate dehydrogenase (LDH) activity was assessed using the lactate dehydrogenase enzyme protection assay. Statistical comparisons were made only between treatments within the same drying condition, as determined by one-way ANOVA followed by Tukey's post hoc test (α = 0.05). Data represent mean ± SE from three independent replicates per condition.

bioRxiv preprint doi: https://doi.org/10.64898/2026.01.16.700019; this version posted January 17, 2026. The copyright holder for this preprint (which was not certified by peer review) is the author/funder, who has granted bioRxiv a license to display the preprint in perpetuity. It is made available under aCC-BY 4.0 International license.

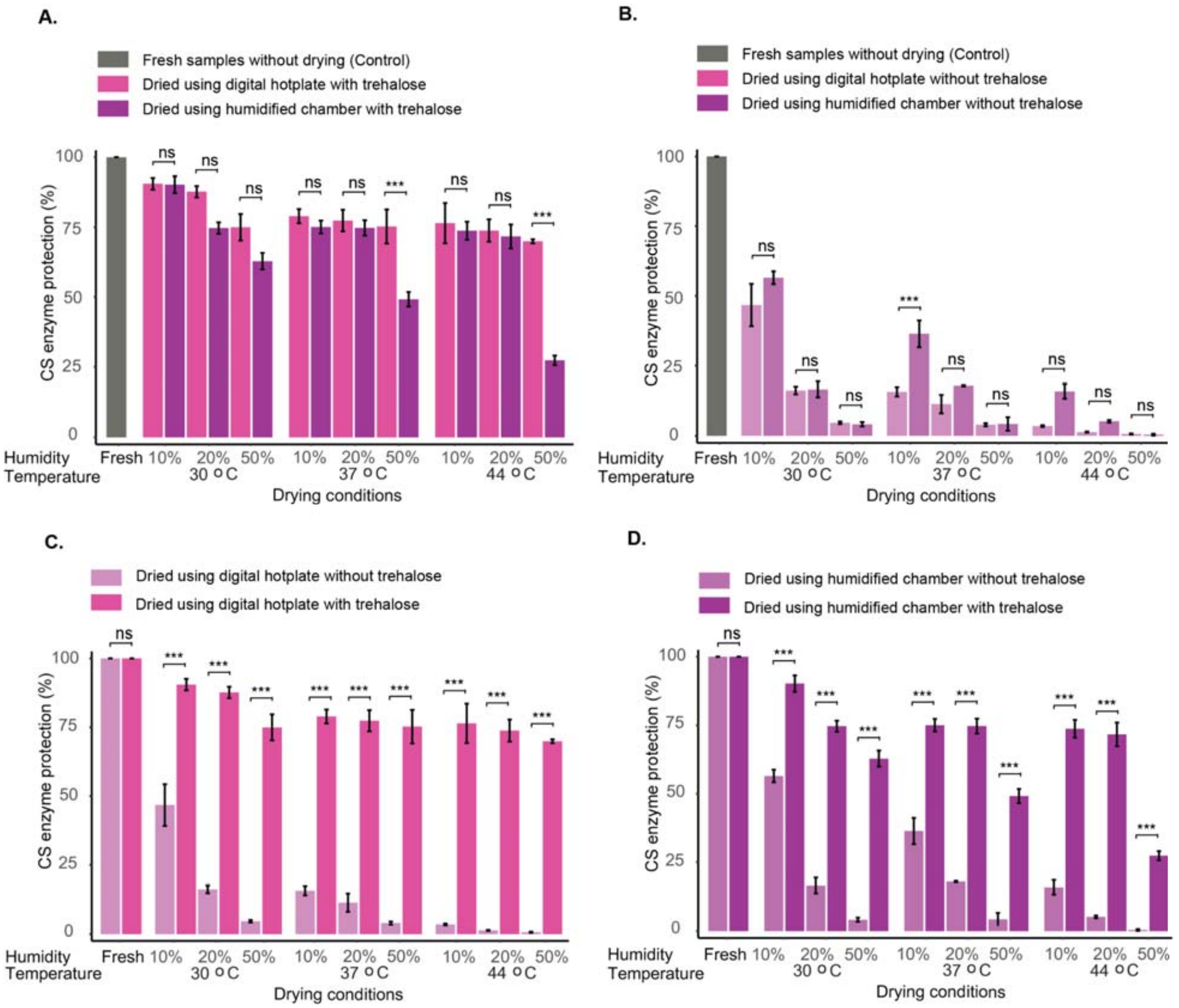

Supplementary Figure S6. Effect of drying method and trehalose on CS activity. Comparison of drying methods with trehalose (A). Comparison of drying methods without trehalose (B). Hotplate drying with and without trehalose (C). Humidified chamber drying with and without trehalose (D). Citrate synthase (CS) activity was assessed using the citrate synthase enzyme protection assay. Statistical comparisons were made only between treatments within the same drying condition, as determined by one-way ANOVA followed by Tukey's post hoc test (α = 0.05). Data represent mean ± SE from three independent replicates per condition.

bioRxiv preprint doi: https://doi.org/10.64898/2026.01.16.700019; this version posted January 17, 2026. The copyright holder for this preprint (which was not certified by peer review) is the author/funder, who has granted bioRxiv a license to display the preprint in perpetuity. It is made available under aCC-BY 4.0 International license.

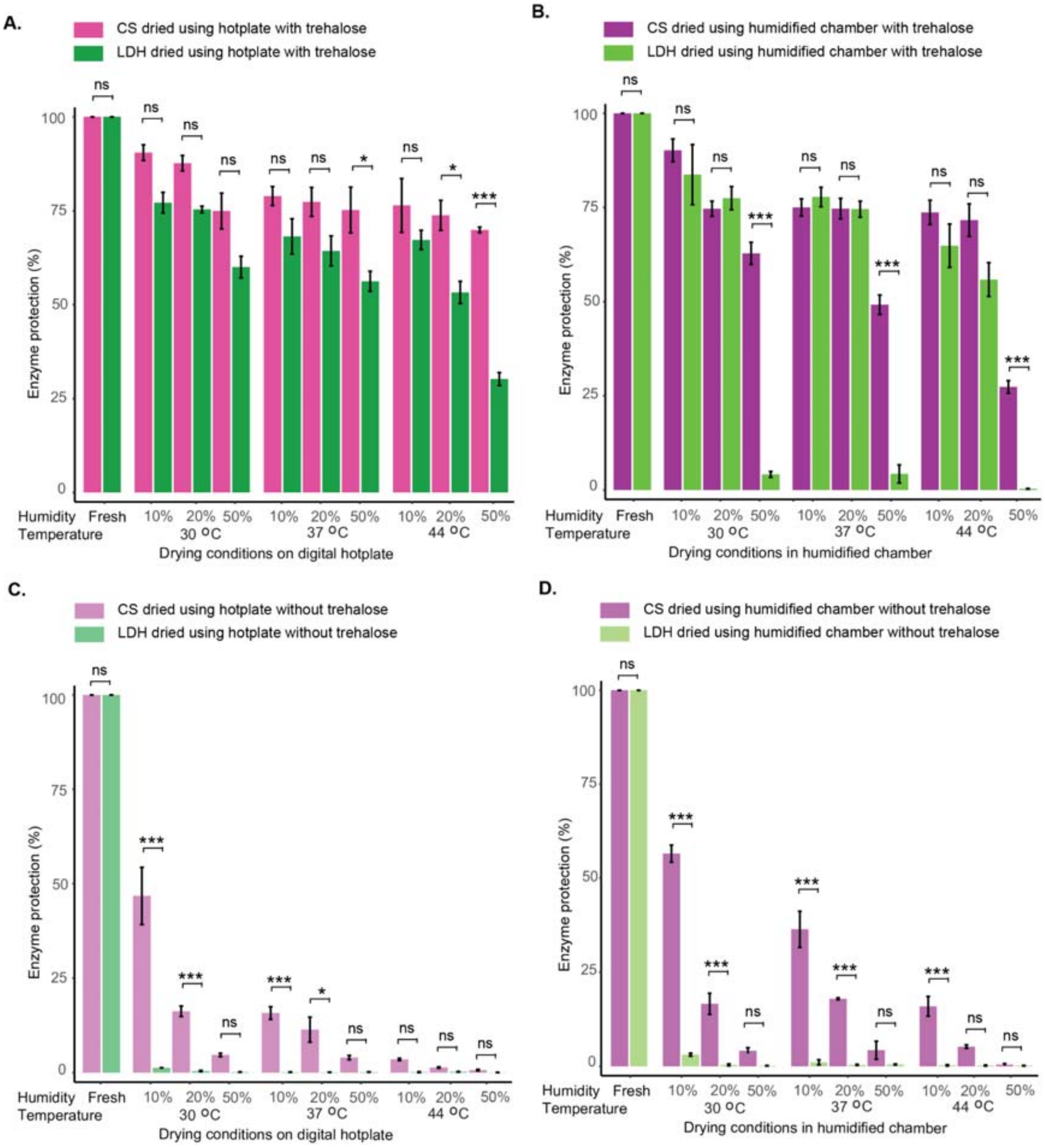

Supplementary Figure S7. Effect of drying method and trehalose on CS and LDH activity. Citrate synthase (CS) and lactate dehydrogenase (LDH) activity in hotplate-dried samples with trehalose (A), humidified chamber-dried samples with trehalose (B), hotplate-dried samples without trehalose (C), and humidified chamber-dried samples without trehalose (D). Activity of CS and LDH was assessed using the citrate synthase and lactate dehydrogenase enzyme protection assays, respectively. Statistical comparisons were made only between treatments within the same drying method, as determined by one-way ANOVA followed by Tukey's post hoc test (α = 0.05). Data represent mean ± SE from three independent replicates per condition.

bioRxiv preprint doi: https://doi.org/10.64898/2026.01.16.700019; this version posted January 17, 2026. The copyright holder for this preprint (which was not certified by peer review) is the author/funder, who has granted bioRxiv a license to display the preprint in perpetuity. It is made available under aCC-BY 4.0 International license.

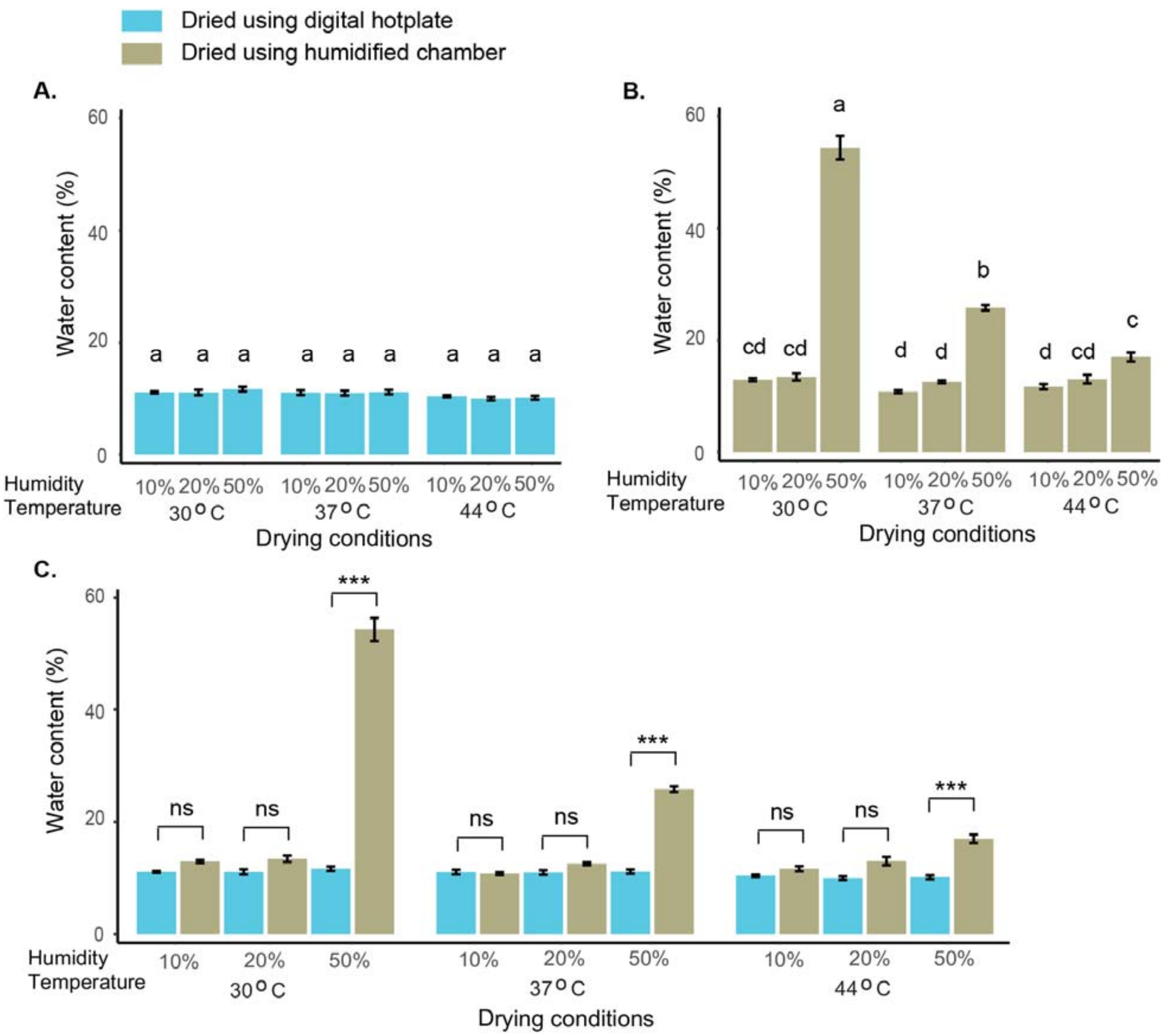

Supplementary Figure S8. Effect of drying methods and conditions on water content in dried samples.

Water content in hotplate-dried samples (A). Water content in humidified chamber-dried samples (B). Comparison of water content between hotplate and humidified chamber drying at each temperature and relative humidity (C). Statistical comparisons in (A) and (B) were determined using one-way ANOVA followed by Tukey's post hoc test (α = 0.05); different letters indicate statistically significant differences among drying conditions within each panel. For (C), statistical comparisons were made only between treatments within the same drying condition using one-way ANOVA followed by Tukey's post hoc test (α = 0.05). Data represent mean ± SE from three independent replicates per condition.

bioRxiv preprint doi: https://doi.org/10.64898/2026.01.16.700019; this version posted January 17, 2026. The copyright holder for this preprint (which was not certified by peer review) is the author/funder, who has granted bioRxiv a license to display the preprint in perpetuity. It is made available under aCC-BY 4.0 International license.

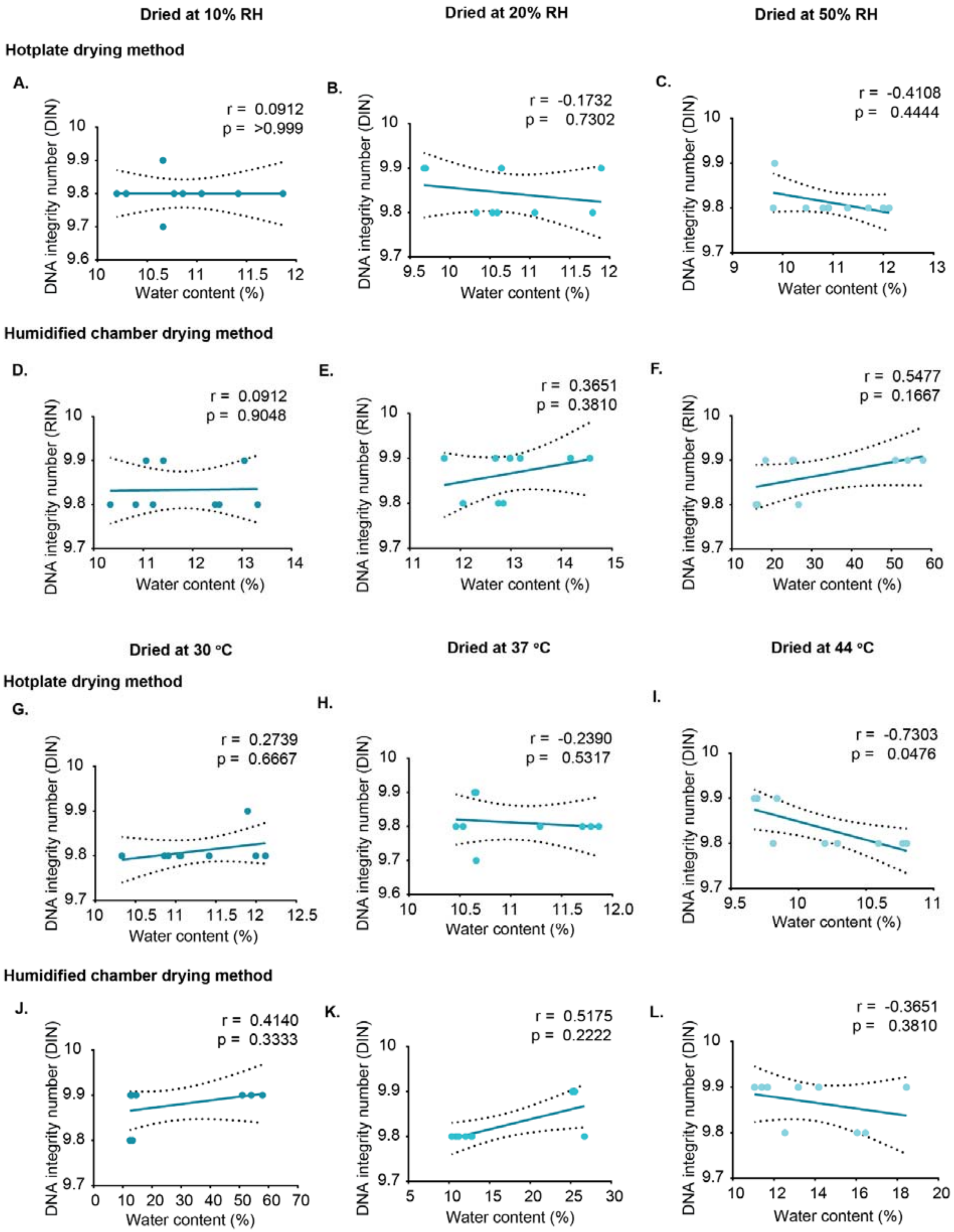

Supplementary Figure S9. Correlation between residual water content and DNA integrity in trehalose-containing samples under constant humidity and constant temperature conditions using two drying methods.

bioRxiv preprint doi: https://doi.org/10.64898/2026.01.16.700019; this version posted January 17, 2026. The copyright holder for this preprint (which was not certified by peer review) is the author/funder, who has granted bioRxiv a license to display the preprint in perpetuity. It is made available under aCC-BY 4.0 International license.

DNA integrity was assessed using the DNA Integrity Number (DIN). A-C show hotplate-dried samples at constant humidity, and D-F show humidified chamber-dried samples at constant humidity. G-I show hotplate-dried samples at constant temperature, and panels J-L show humidified chamber-dried samples at constant temperature. Correlation coefficients (r) and significance values (p) were calculated using Pearson correlation for normally distributed data and Spearman correlation for non-normally distributed data. Each data point represents an individual replicate. Dashed lines indicate 95% confidence interval (CI).

bioRxiv preprint doi: https://doi.org/10.64898/2026.01.16.700019; this version posted January 17, 2026. The copyright holder for this preprint (which was not certified by peer review) is the author/funder, who has granted bioRxiv a license to display the preprint in perpetuity. It is made available under aCC-BY 4.0 International license.

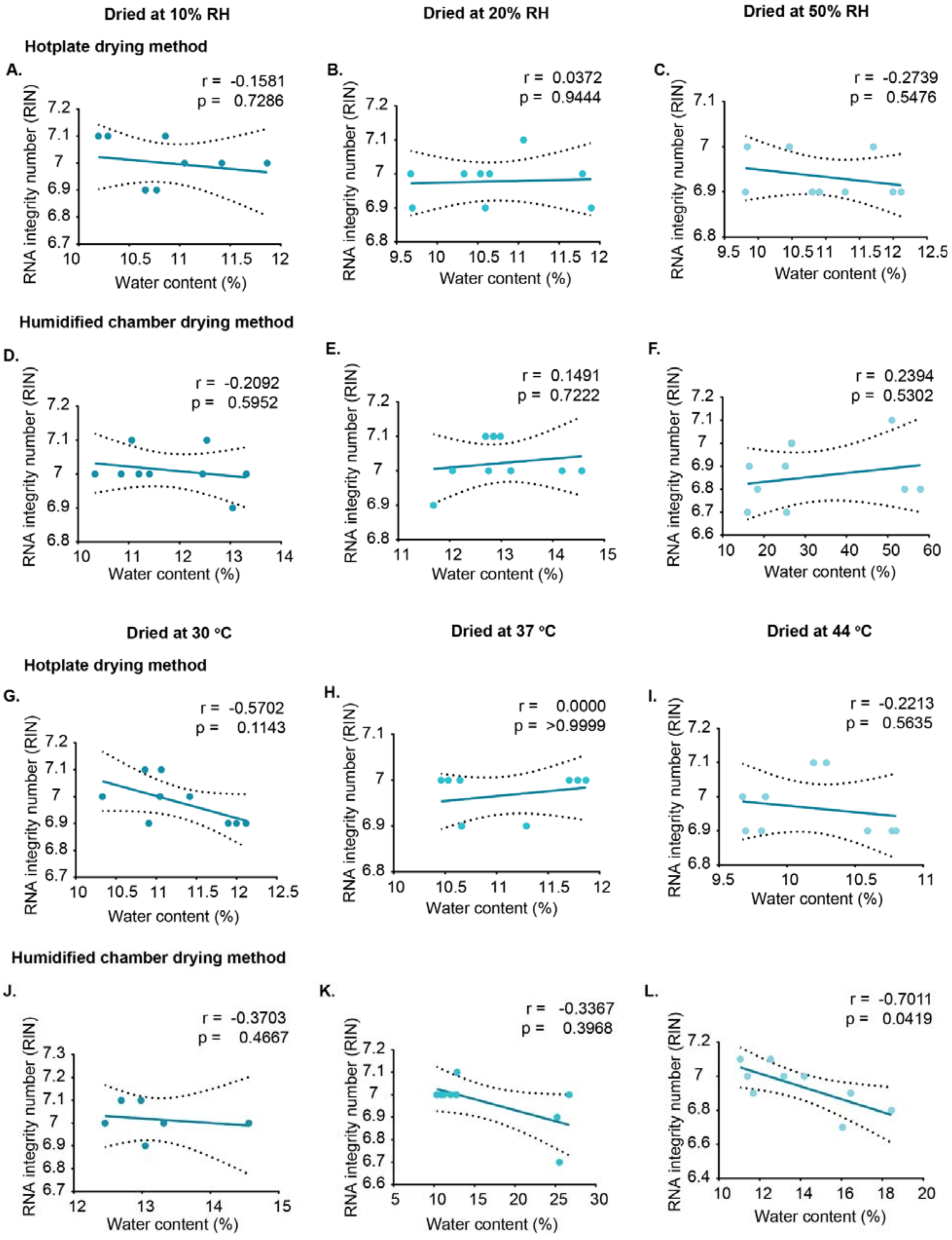

bioRxiv preprint doi: https://doi.org/10.64898/2026.01.16.700019; this version posted January 17, 2026. The copyright holder for this preprint (which was not certified by peer review) is the author/funder, who has granted bioRxiv a license to display the preprint in perpetuity. It is made available under aCC-BY 4.0 International license.

Supplementary Figure S10. Correlation between residual water content and RNA integrity in trehalose-containing samples under constant humidity and constant temperature conditions using two drying methods.
RNA integrity was assessed using the RNA Integrity Number (RIN). A-C show hotplate-dried samples at constant humidity, and D-F show humidified chamber-dried samples at constant humidity. G-I show hotplate-dried samples at constant temperature, and J-L show humidified chamber-dried samples at constant temperature. Correlation coefficients (r) and significance values (p) were calculated using Pearson correlation for normally distributed data and Spearman correlation for non-normally distributed data. Each data point represents an individual replicate. Dashed lines indicate 95% confidence interval (CI).

bioRxiv preprint doi: https://doi.org/10.64898/2026.01.16.700019; this version posted January 17, 2026. The copyright holder for this preprint (which was not certified by peer review) is the author/funder, who has granted bioRxiv a license to display the preprint in perpetuity. It is made available under aCC-BY 4.0 International license.

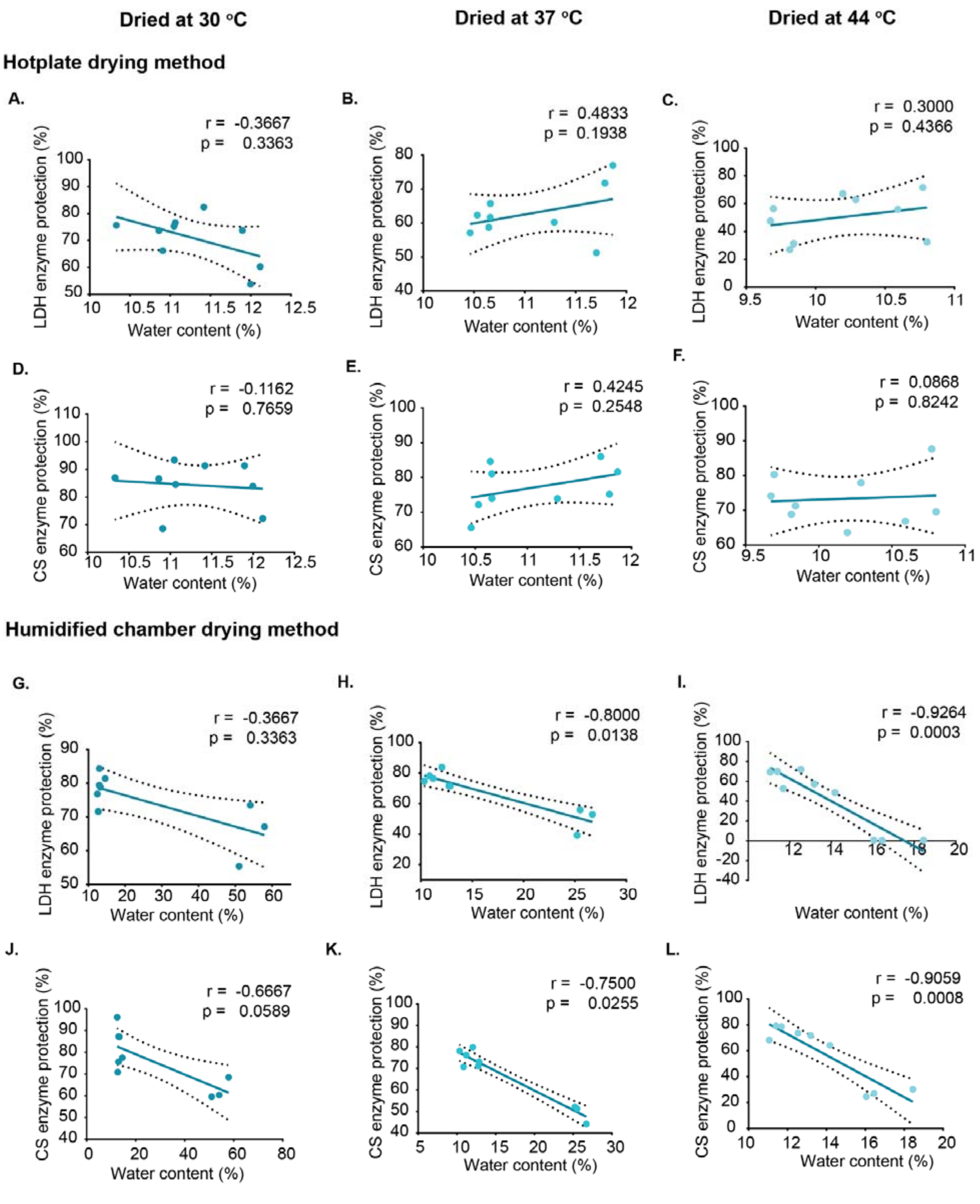

Supplementary Figure S11. Correlation between residual water content and enzyme activity of LDH and CS under constant temperature and varying humidity conditions using two drying methods.

bioRxiv preprint doi: https://doi.org/10.64898/2026.01.16.700019; this version posted January 17, 2026. The copyright holder for this preprint (which was not certified by peer review) is the author/funder, who has granted bioRxiv a license to display the preprint in perpetuity. It is made available under aCC-BY 4.0 International license.

Enzymatic activity of lactate dehydrogenase (LDH; A-C, G-I) and citrate synthase (CS; D-F, J-L) was assessed after drying under three different temperatures at each of the following constant relative humidity levels: 10%, 20%, and 50%. Drying was performed using either the hotplate method (A-F) or the humidified chamber method (G-L). Correlation coefficients (r) and significance values (p) were calculated using Pearson correlation for normally distributed data and Spearman correlation for non-normally distributed data. Each data point represents an individual replicate. Dashed lines indicate 95% confidence interval (CI).

bioRxiv preprint doi: https://doi.org/10.64898/2026.01.16.700019; this version posted January 17, 2026. The copyright holder for this preprint (which was not certified by peer review) is the author/funder, who has granted bioRxiv a license to display the preprint in perpetuity. It is made available under aCC-BY 4.0 International license.

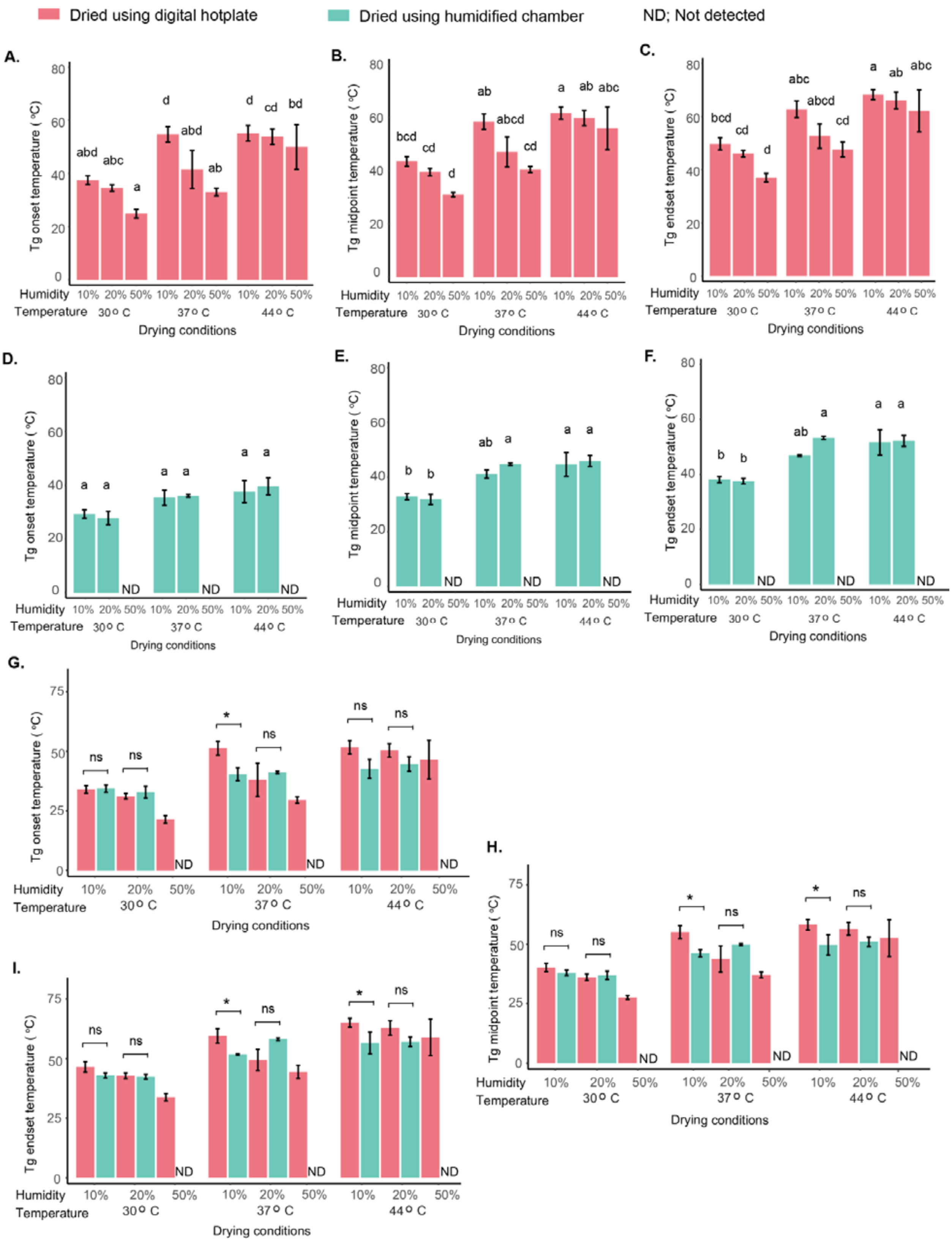

bioRxiv preprint doi: https://doi.org/10.64898/2026.01.16.700019; this version posted January 17, 2026. The copyright holder for this preprint (which was not certified by peer review) is the author/funder, who has granted bioRxiv a license to display the preprint in perpetuity. It is made available under aCC-BY 4.0 International license.

Supplementary Figure S12. Glass transition temperature (Tg) of trehalose-containing samples under different drying methods and environmental conditions.
Tg onset (A, D, G), midpoint (B, E, H), and endset (C, F, I) were measured for samples dried using the hotplate method (A-C) or the humidified chamber method (panels D-F) across a range of temperatures and relative humidities. G-I show direct comparisons of Tg values between hotplate and humidified chamber drying at each temperature and relative humidity. Statistical comparisons in A-F were determined using one-way ANOVA followed by Tukey's post hoc test ($\alpha = 0.05$); different letters indicate statistically significant differences among drying conditions within each panel. For G-I, statistical comparisons were made only between treatments within the same drying condition. Data represent mean ± SE from three independent replicates per condition.

bioRxiv preprint doi: https://doi.org/10.64898/2026.01.16.700019; this version posted January 17, 2026. The copyright holder for this preprint (which was not certified by peer review) is the author/funder, who has granted bioRxiv a license to display the preprint in perpetuity. It is made available under aCC-BY 4.0 International license.

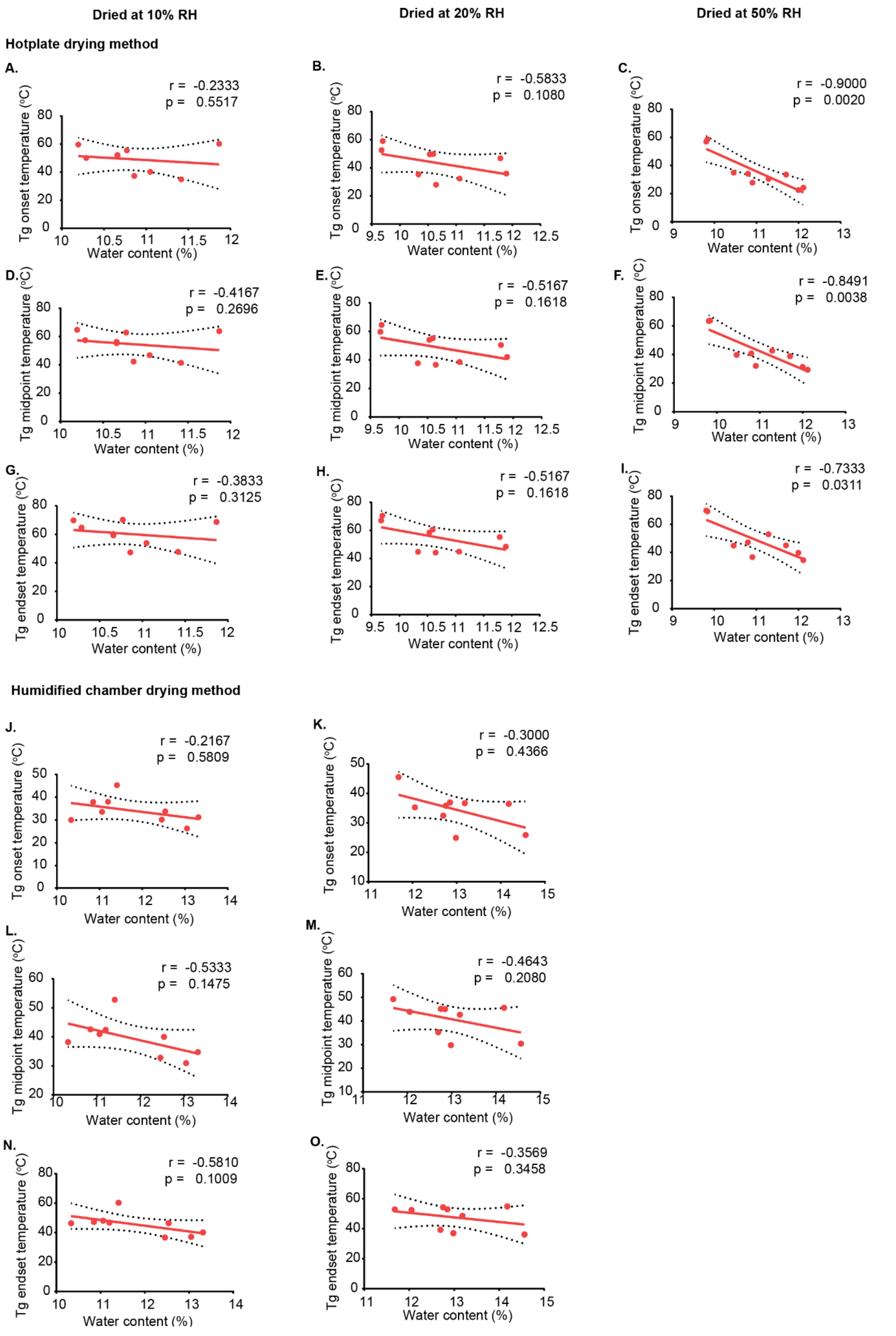

bioRxiv preprint doi: https://doi.org/10.64898/2026.01.16.700019; this version posted January 17, 2026. The copyright holder for this preprint (which was not certified by peer review) is the author/funder, who has granted bioRxiv a license to display the preprint in perpetuity. It is made available under aCC-BY 4.0 International license.

Supplementary Figure S13. Correlation between residual water content and glass transition temperature (Tg) in trehalose-containing samples.

Tg onset (A-C, J-K), midpoint (D-F, L-M), and endset (G-I, N-O) were assessed for samples dried under constant humidity with varying temperatures using either the hotplate method (A-I) or the humidified chamber method (J-O). Correlation coefficients (r) and significance values (p) were calculated using Pearson correlation for normally distributed data and Spearman correlation for non-normally distributed data. Each data point represents an individual replicate. Dashed lines indicate 95% confidence interval (CI).

bioRxiv preprint doi: https://doi.org/10.64898/2026.01.16.700019; this version posted January 17, 2026. The copyright holder for this preprint (which was not certified by peer review) is the author/funder, who has granted bioRxiv a license to display the preprint in perpetuity. It is made available under aCC-BY 4.0 International license.

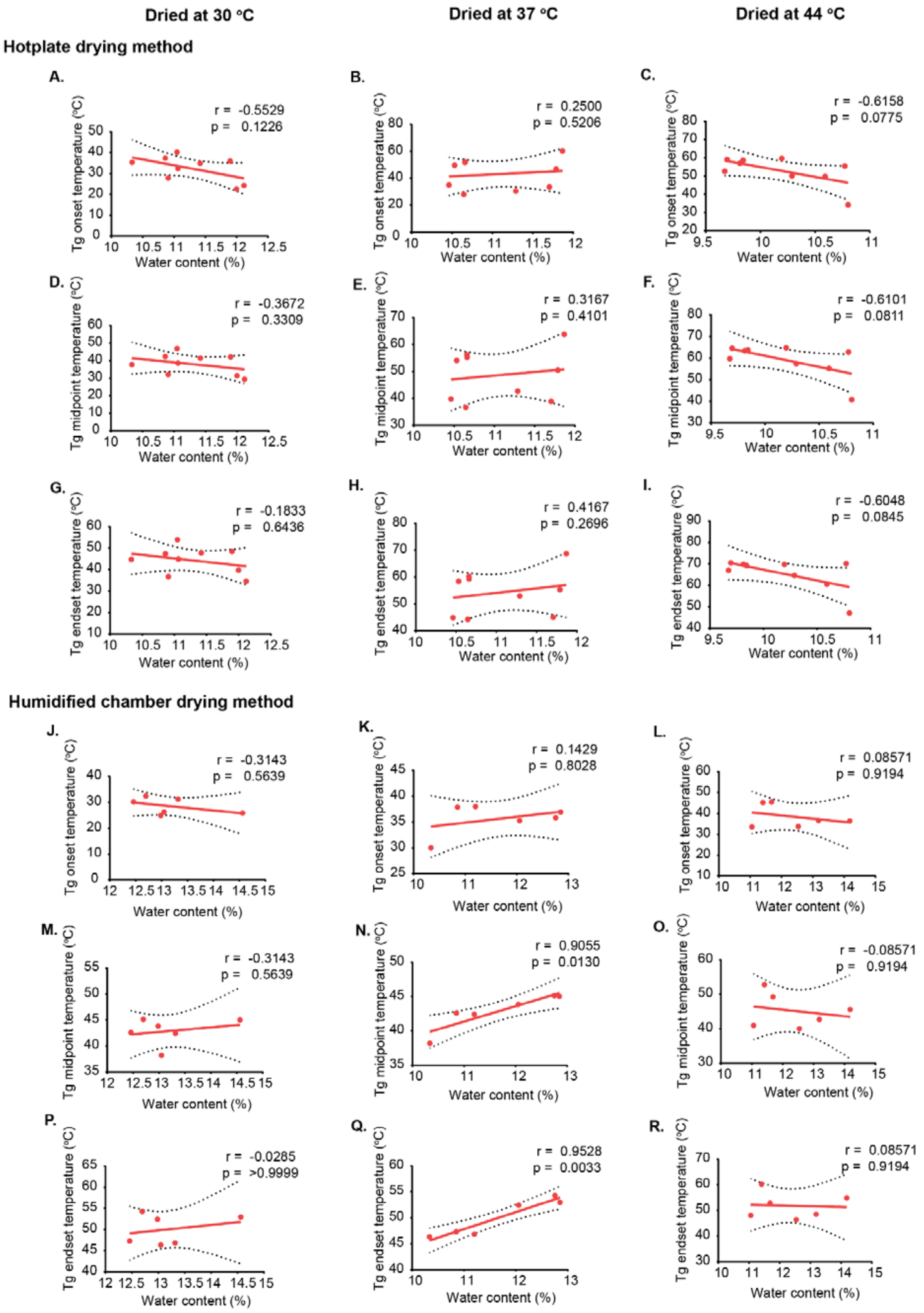

bioRxiv preprint doi: https://doi.org/10.64898/2026.01.16.700019; this version posted January 17, 2026. The copyright holder for this preprint (which was not certified by peer review) is the author/funder, who has granted bioRxiv a license to display the preprint in perpetuity. It is made available under aCC-BY 4.0 International license.

Supplementary Figure S14. Correlation between residual water content and glass transition temperature (Tg) in trehalose-containing samples.

Tg onset (A-C, J-L), midpoint (D-F, M-O), and endset (G-I, P-R) were assessed for samples dried under constant temperature with varying relative humidities using either the hotplate method (A-I) or the humidified chamber method (J-R). Correlation coefficients (r) and significance values (p) were calculated using Pearson correlation for normally distributed data and Spearman correlation for non-normally distributed data. Each data point represents an individual replicate. Dashed lines indicate 95% confidence interval (CI).

bioRxiv preprint doi: https://doi.org/10.64898/2026.01.16.700019; this version posted January 17, 2026. The copyright holder for this preprint (which was not certified by peer review) is the author/funder, who has granted bioRxiv a license to display the preprint in perpetuity. It is made available under aCC-BY 4.0 International license.

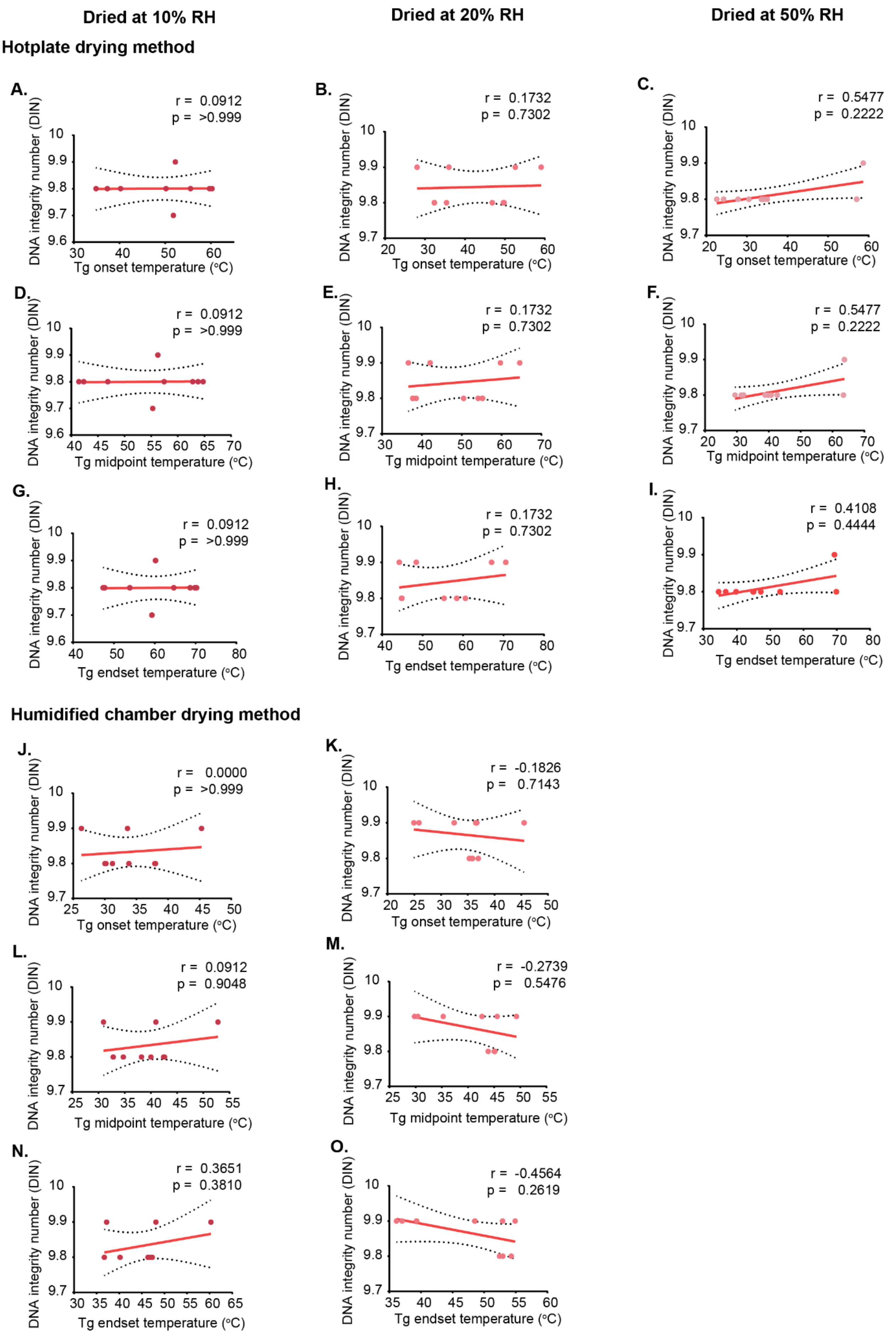

bioRxiv preprint doi: https://doi.org/10.64898/2026.01.16.700019; this version posted January 17, 2026. The copyright holder for this preprint (which was not certified by peer review) is the author/funder, who has granted bioRxiv a license to display the preprint in perpetuity. It is made available under aCC-BY 4.0 International license.

Supplementary Figure S15. Correlation between glass transition temperature (Tg) and DNA integrity in samples dried using the hotplate and humidified chamber methods under constant humidity conditions.

DNA integrity was assessed using the DNA Integrity Number (DIN). A-I show correlations between Tg values (onset, midpoint, and endset) and DNA integrity for hotplate-dried samples at 10%, 20%, and 50% relative humidity and humidified chamber-dried samples at 10% and 20% RH (50% RH not included for humidified chamber due to the absence of distinct Tg). Correlation coefficients (r) and significance values (p) were calculated using Pearson correlation for normally distributed data and Spearman correlation for non-normally distributed data. Each data point represents an individual replicate. Dashed lines indicate 95% confidence interval (CI).

bioRxiv preprint doi: https://doi.org/10.64898/2026.01.16.700019; this version posted January 17, 2026. The copyright holder for this preprint (which was not certified by peer review) is the author/funder, who has granted bioRxiv a license to display the preprint in perpetuity. It is made available under aCC-BY 4.0 International license.

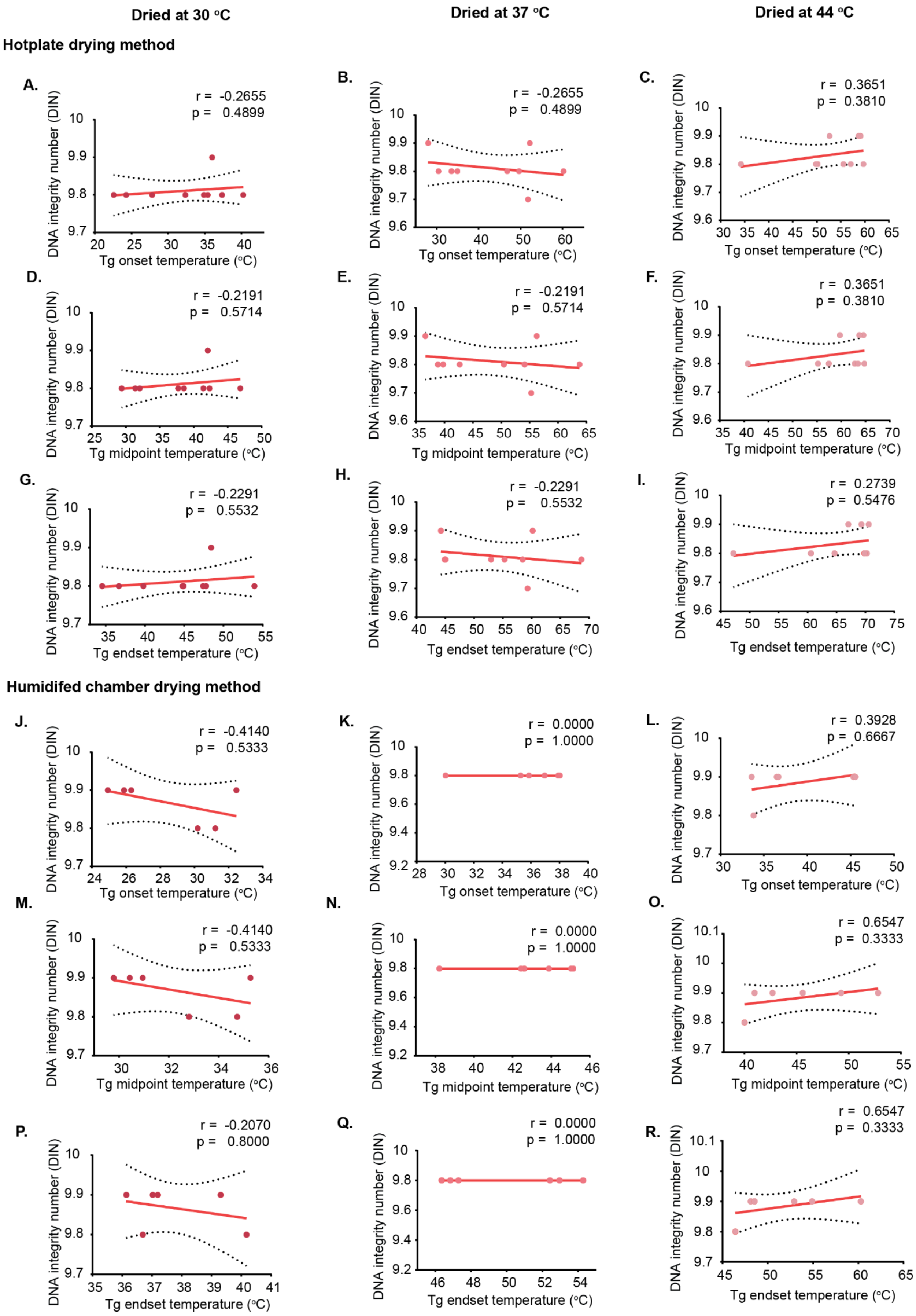

bioRxiv preprint doi: https://doi.org/10.64898/2026.01.16.700019; this version posted January 17, 2026. The copyright holder for this preprint (which was not certified by peer review) is the author/funder, who has granted bioRxiv a license to display the preprint in perpetuity. It is made available under aCC-BY 4.0 International license.

Supplementary Figure S16. Correlation between glass transition temperature (Tg) and DNA integrity in samples dried using the hotplate and humidified chamber methods under constant temperature conditions.

DNA integrity was assessed using the DNA Integrity Number (DIN). A-I show correlations between Tg values (onset, midpoint, and endset) and DNA integrity for hotplate-dried samples at 30 °C, 37 °C, and 44 °C, while J-R show correlations for humidified chamber-dried samples at the same temperatures. Correlation coefficients (r) and significance values (p) were calculated using Pearson correlation for normally distributed data and Spearman correlation for non-normally distributed data. Each data point represents an individual replicate. Dashed lines indicate 95% confidence interval (CI).

bioRxiv preprint doi: https://doi.org/10.64898/2026.01.16.700019; this version posted January 17, 2026. The copyright holder for this preprint (which was not certified by peer review) is the author/funder, who has granted bioRxiv a license to display the preprint in perpetuity. It is made available under aCC-BY 4.0 International license.

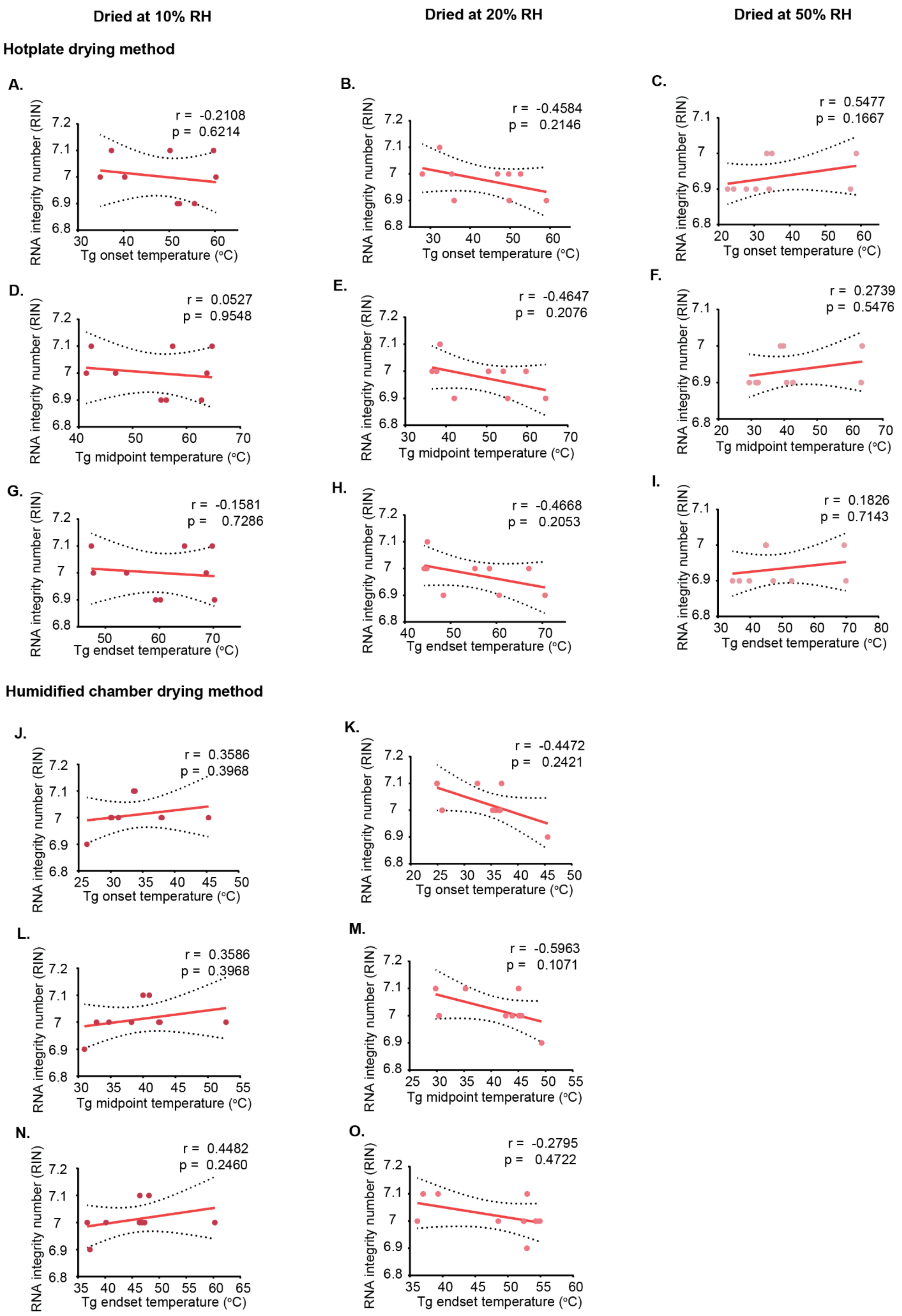

bioRxiv preprint doi: https://doi.org/10.64898/2026.01.16.700019; this version posted January 17, 2026. The copyright holder for this preprint (which was not certified by peer review) is the author/funder, who has granted bioRxiv a license to display the preprint in perpetuity. It is made available under aCC-BY 4.0 International license.

Supplementary Figure S17. Correlation between glass transition temperature (Tg) and RNA integrity in samples dried using the hotplate and humidified chamber methods under constant humidity conditions.

RNA integrity was assessed using the RNA Integrity Number (RIN). A-I show correlations between Tg values (onset, midpoint, and endset) and RNA integrity for hotplate-dried samples at 10%, 20%, and 50% relative humidity and humidified chamber-dried samples at 10% and 20% RH (50% RH not included for humidified chamber due to the absence of distinct Tg). Correlation coefficients (r) and significance values (p) were calculated using Pearson correlation for normally distributed data and Spearman correlation for non-normally distributed data. Each data point represents an individual replicate. Dashed lines indicate 95% confidence interval (CI).

bioRxiv preprint doi: https://doi.org/10.64898/2026.01.16.700019; this version posted January 17, 2026. The copyright holder for this preprint (which was not certified by peer review) is the author/funder, who has granted bioRxiv a license to display the preprint in perpetuity. It is made available under aCC-BY 4.0 International license.

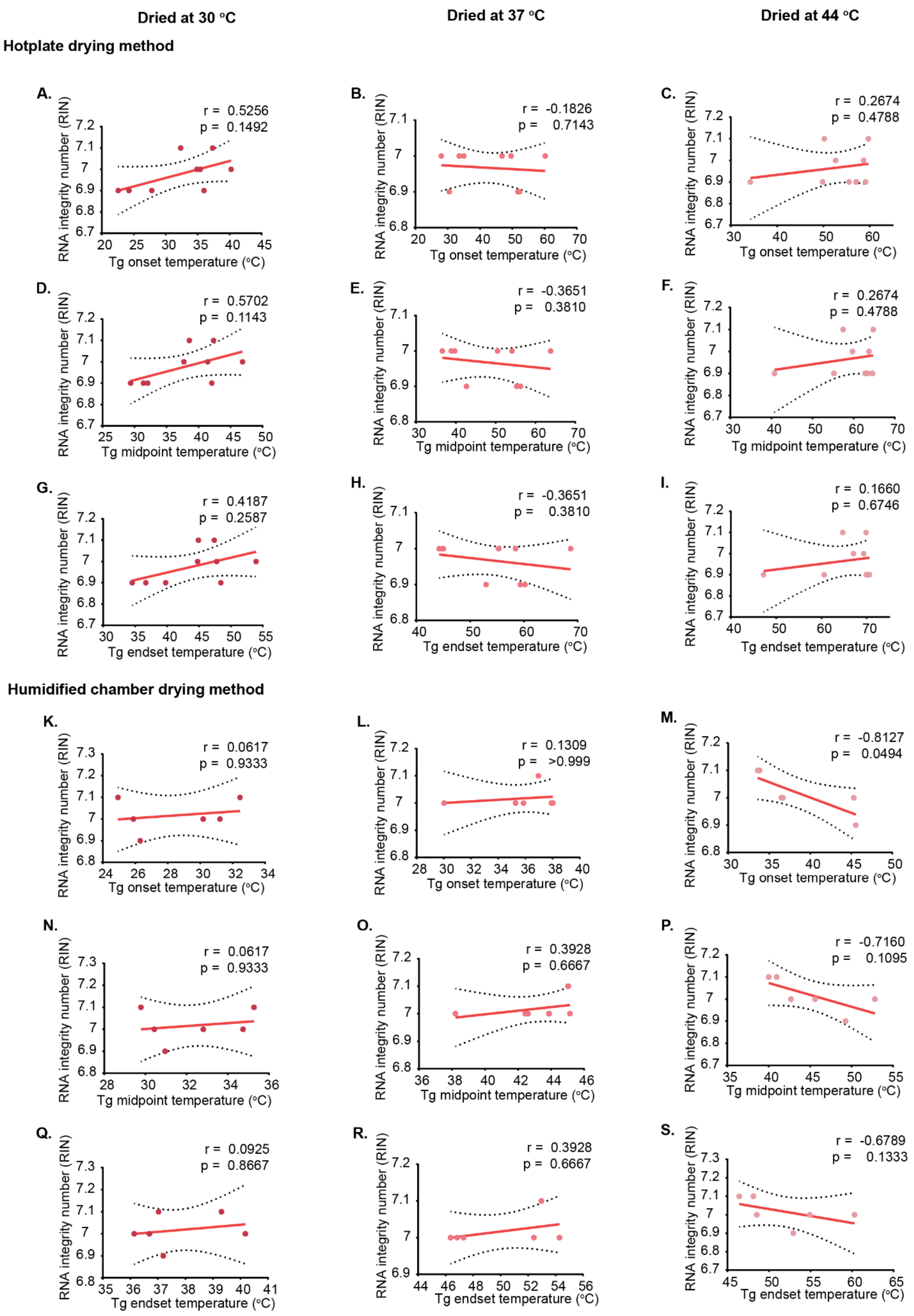

bioRxiv preprint doi: https://doi.org/10.64898/2026.01.16.700019; this version posted January 17, 2026. The copyright holder for this preprint (which was not certified by peer review) is the author/funder, who has granted bioRxiv a license to display the preprint in perpetuity. It is made available under aCC-BY 4.0 International license.

Supplementary Figure S18. Correlation between glass transition temperature (Tg) and RNA integrity in samples dried using the hotplate and humidified chamber methods under constant temperature conditions.

RNA integrity was assessed using the RNA Integrity Number (RIN). A-I show correlations between Tg values (onset, midpoint, and endset) and RNA integrity for hotplate-dried samples at 30 °C, 37 °C, and 44 °C, while J-R show correlations for humidified chamber-dried samples at the same temperatures. Correlation coefficients (r) and significance values (p) were calculated using Pearson correlation for normally distributed data and Spearman correlation for non-normally distributed data. Each data point represents an individual replicate. Dashed lines indicate 95% confidence interval (CI).

bioRxiv preprint doi: https://doi.org/10.64898/2026.01.16.700019; this version posted January 17, 2026. The copyright holder for this preprint (which was not certified by peer review) is the author/funder, who has granted bioRxiv a license to display the preprint in perpetuity. It is made available under aCC-BY 4.0 International license.

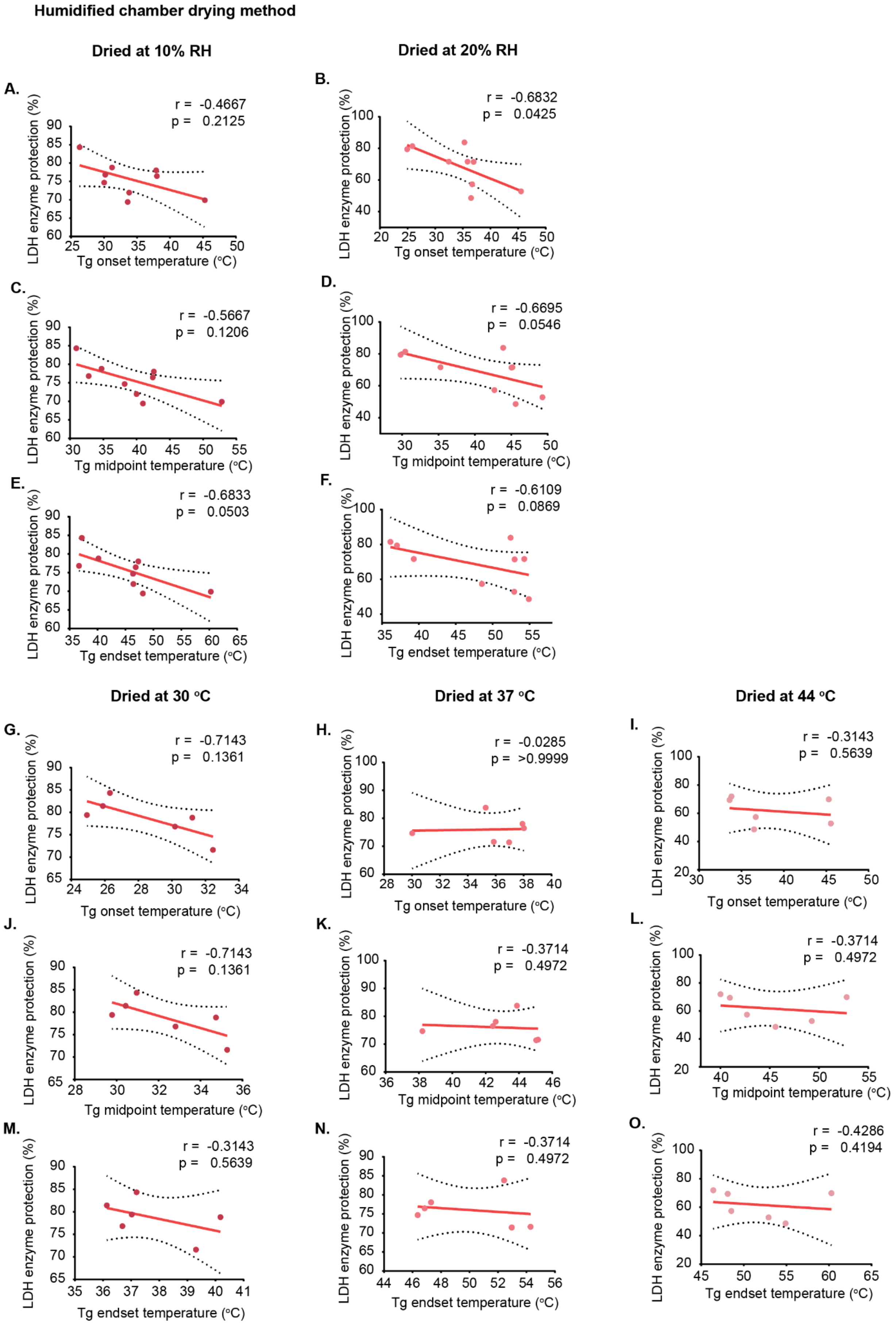

bioRxiv preprint doi: https://doi.org/10.64898/2026.01.16.700019; this version posted January 17, 2026. The copyright holder for this preprint (which was not certified by peer review) is the author/funder, who has granted bioRxiv a license to display the preprint in perpetuity. It is made available under aCC-BY 4.0 International license.

Supplementary Figure S19. Correlation between glass transition temperature (Tg) and LDH activity in samples dried using the humidified chamber method under constant humidity and temperature conditions.
A-F show correlations between Tg values (onset, midpoint, and endset) and lactate dehydrogenase (LDH) activity under constant humidity conditions (10% and 20% RH; 50% RH not included due to absence of measurable Tg), while panels G-O show correlations under constant temperature conditions (30 °C, 37 °C, and 44 °C). Correlation coefficients (r) and significance values (p) were calculated using Pearson correlation for normally distributed data and Spearman correlation for non-normally distributed data. Each data point represents an individual replicate. Dashed lines indicate 95% confidence interval (CI).

bioRxiv preprint doi: https://doi.org/10.64898/2026.01.16.700019; this version posted January 17, 2026. The copyright holder for this preprint (which was not certified by peer review) is the author/funder, who has granted bioRxiv a license to display the preprint in perpetuity. It is made available under aCC-BY 4.0 International license.

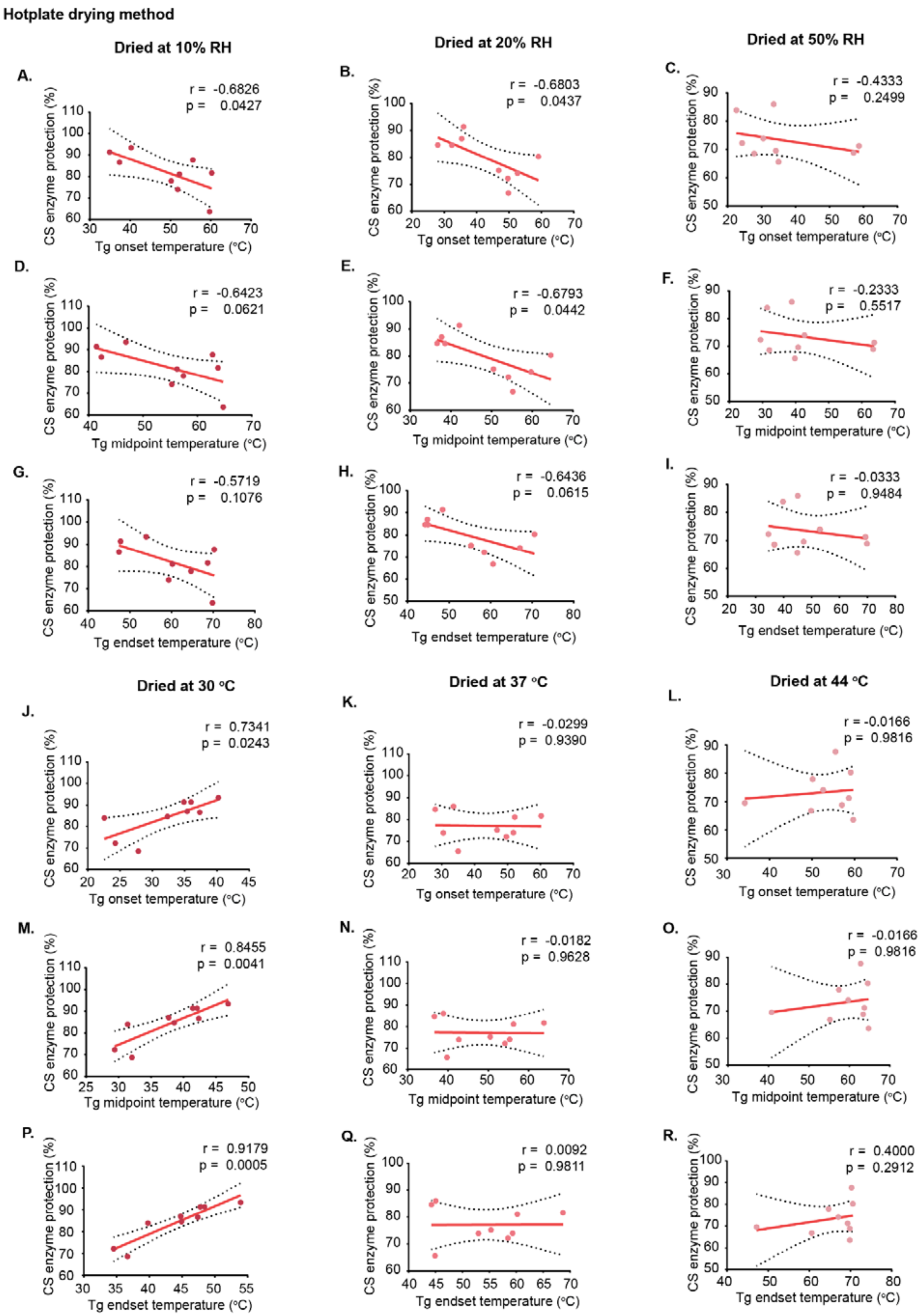

bioRxiv preprint doi: https://doi.org/10.64898/2026.01.16.700019; this version posted January 17, 2026. The copyright holder for this preprint (which was not certified by peer review) is the author/funder, who has granted bioRxiv a license to display the preprint in perpetuity. It is made available under aCC-BY 4.0 International license.

Supplementary Figure S20. Correlation between glass transition temperature (Tg) and CS activity in samples dried using the hotplate method under constant humidity and temperature conditions.

Citrate synthase (CS) activity was assessed in samples dried using the hotplate method. A-I show correlations between Tg values (onset, midpoint, and endset) and CS activity under constant humidity conditions (10%, 20%, and 50% RH), while J-R show correlations under constant temperature conditions (30 °C, 37 °C, and 44 °C). Correlation coefficients (r) and significance values (p) were calculated using Pearson correlation for normally distributed data and Spearman correlation for non-normally distributed data. Each data point represents an individual replicate. Dashed lines indicate 95% confidence interval (CI).

bioRxiv preprint doi: https://doi.org/10.64898/2026.01.16.700019; this version posted January 17, 2026. The copyright holder for this preprint (which was not certified by peer review) is the author/funder, who has granted bioRxiv a license to display the preprint in perpetuity. It is made available under aCC-BY 4.0 International license.

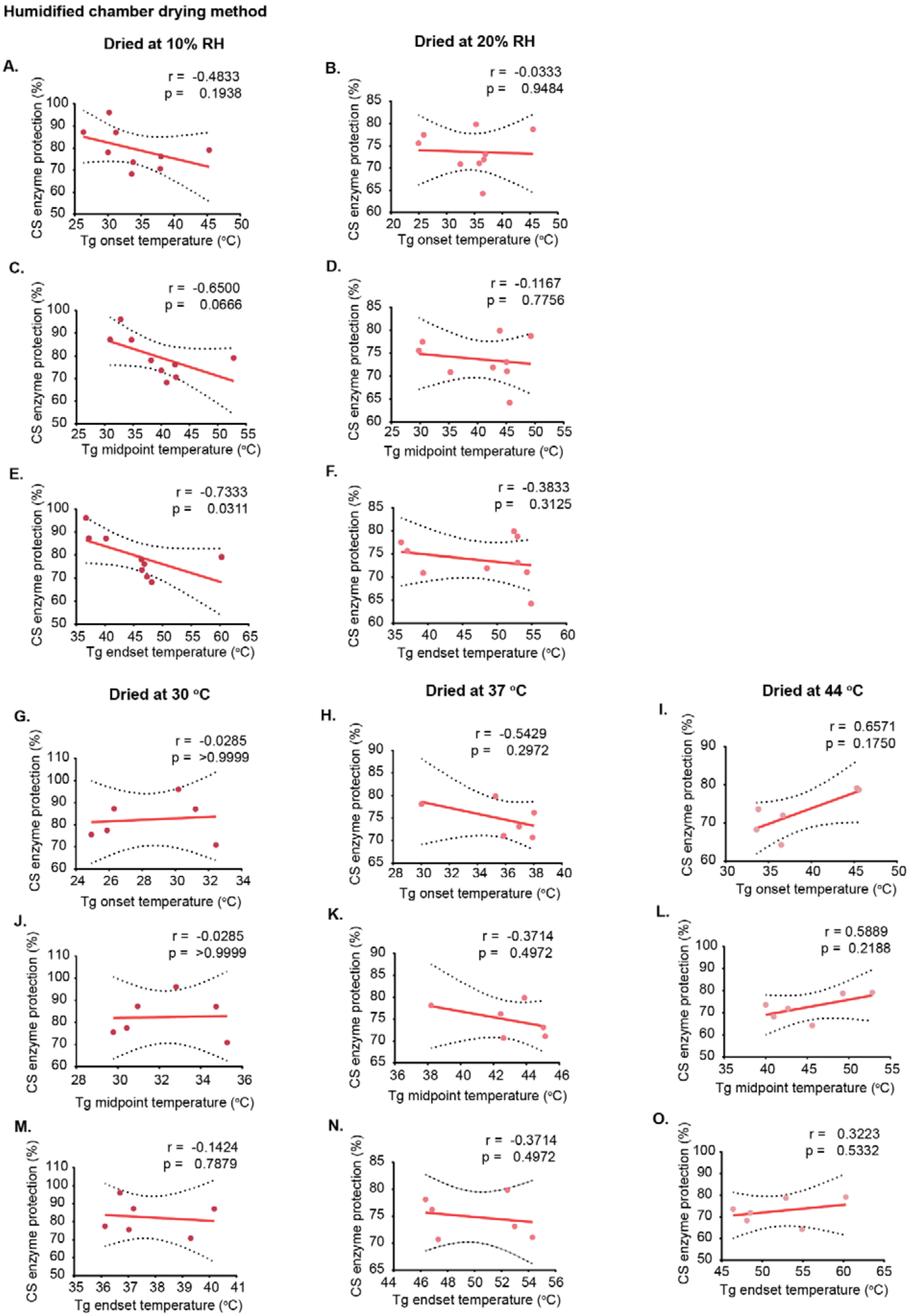

bioRxiv preprint doi: https://doi.org/10.64898/2026.01.16.700019; this version posted January 17, 2026. The copyright holder for this preprint (which was not certified by peer review) is the author/funder, who has granted bioRxiv a license to display the preprint in perpetuity. It is made available under aCC-BY 4.0 International license.

Supplementary Figure S21. Correlation between glass transition temperature (Tg) and CS activity in samples dried using the humidified chamber method under constant humidity and temperature conditions.

A-F show correlations between Tg values (onset, midpoint, and endset) and CS activity under constant humidity conditions (10% and 20% RH; 50% RH not included due to absence of measurable Tg), while G-O show correlations under constant temperature conditions (30 °C, 37 °C, and 44 °C). Correlation coefficients (r) and significance values (p) were calculated using Pearson correlation for normally distributed data and Spearman correlation for non-normally distributed data. Each data point represents an individual replicate. Dashed lines indicate 95% confidence interval (CI).

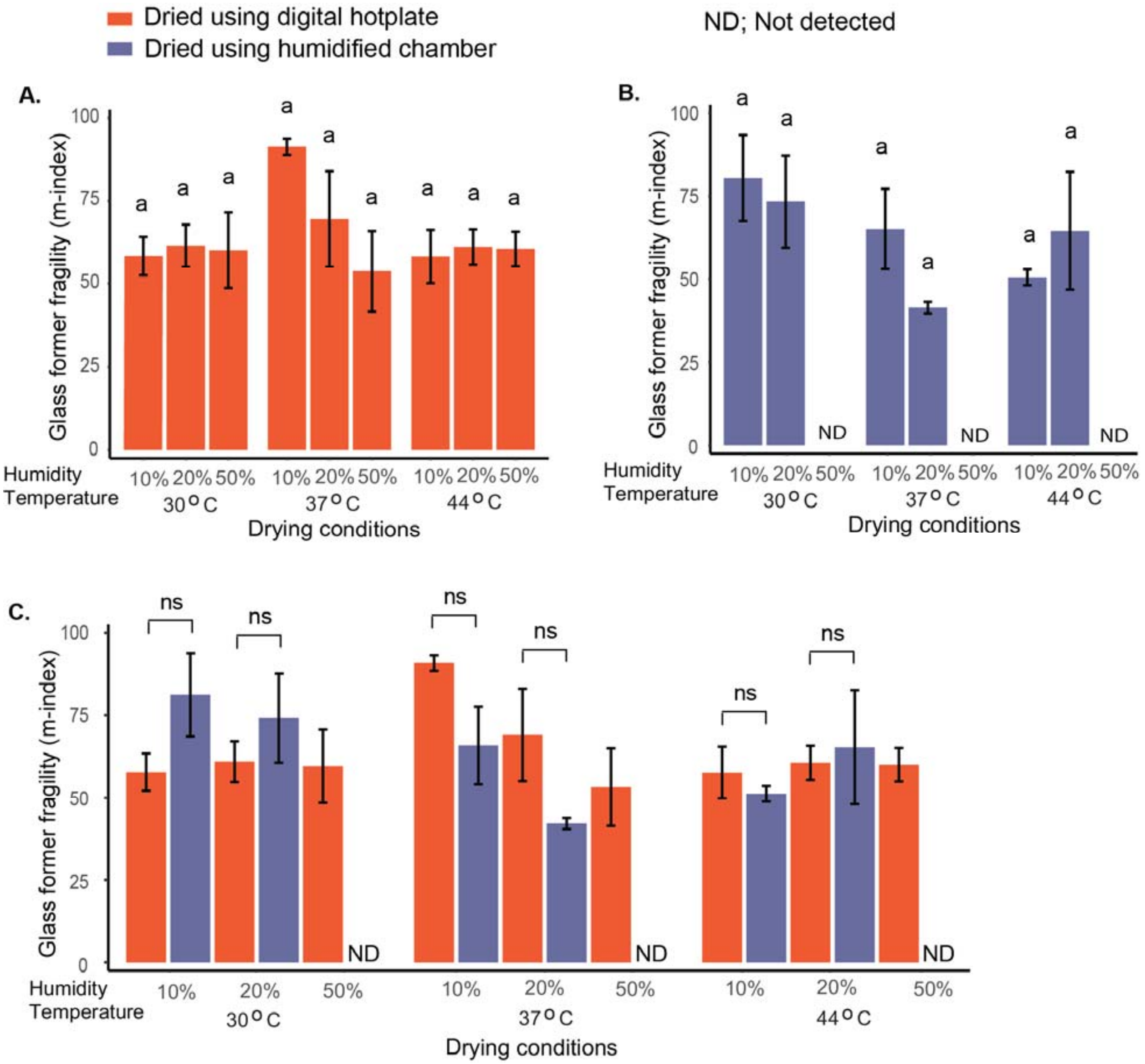

Supplementary Figure S22. Effect of drying methods and conditions on glass former fragility (m-index) in dried samples.

Glass former fragility (m-index) in hotplate-dried samples (A) and humidified chamber-dried samples (B). Comparison of fragility between hotplate and humidified chamber drying at each

bioRxiv preprint doi: https://doi.org/10.64898/2026.01.16.700019; this version posted January 17, 2026. The copyright holder for this preprint (which was not certified by peer review) is the author/funder, who has granted bioRxiv a license to display the preprint in perpetuity. It is made available under aCC-BY 4.0 International license.

temperature and relative humidity (C). Statistical comparisons in panels A and B were performed using one-way ANOVA followed by Tukey's post hoc test (α = 0.05); different letters indicate statistically significant differences among drying conditions within each panel. For panel C, comparisons were made only between treatments within the same drying condition using one-way ANOVA followed by Tukey's post hoc test (α = 0.05). Data represent mean ± SE from three independent replicates per condition.

bioRxiv preprint doi: https://doi.org/10.64898/2026.01.16.700019; this version posted January 17, 2026. The copyright holder for this preprint (which was not certified by peer review) is the author/funder, who has granted bioRxiv a license to display the preprint in perpetuity. It is made available under aCC-BY 4.0 International license.

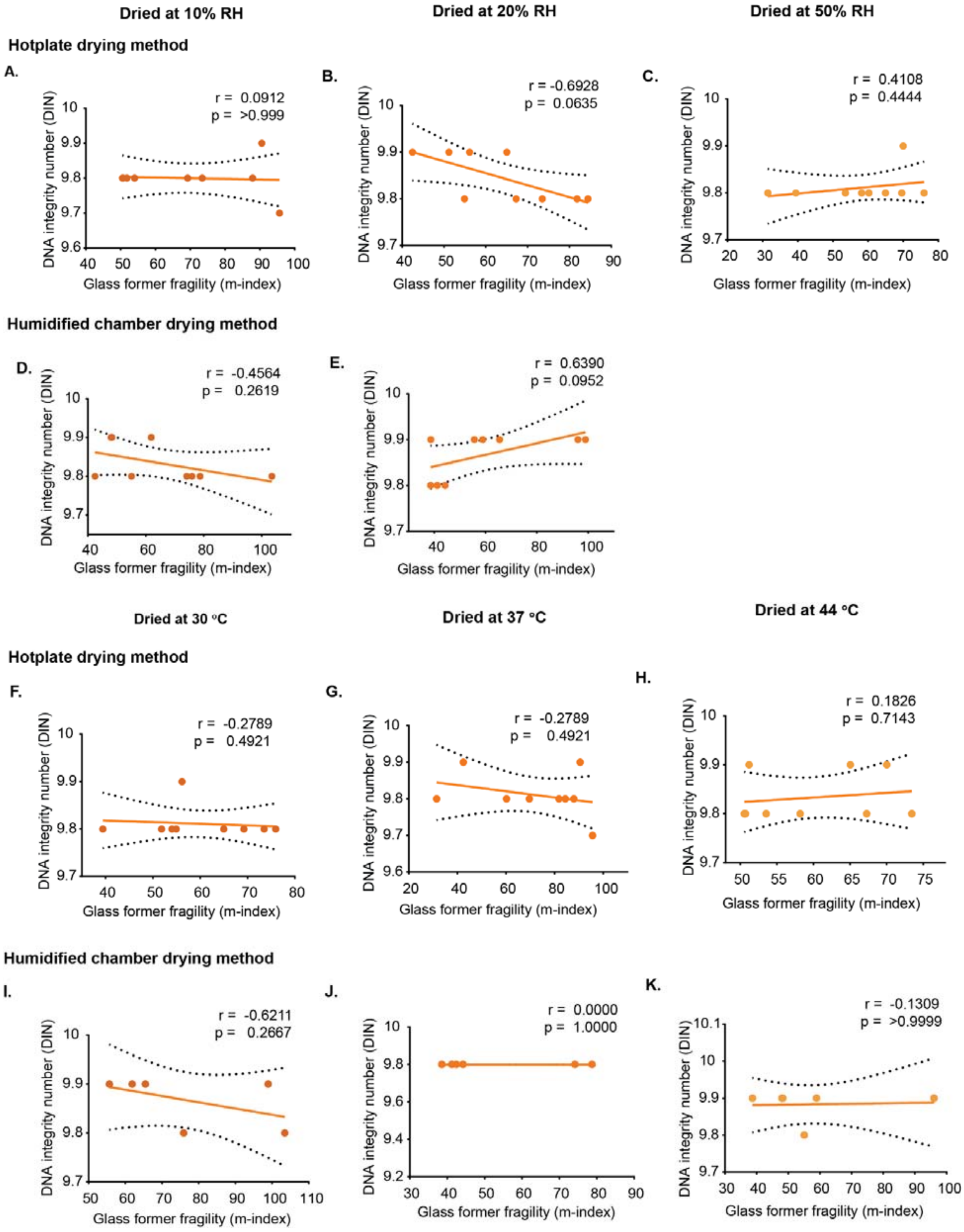

bioRxiv preprint doi: https://doi.org/10.64898/2026.01.16.700019; this version posted January 17, 2026. The copyright holder for this preprint (which was not certified by peer review) is the author/funder, who has granted bioRxiv a license to display the preprint in perpetuity. It is made available under aCC-BY 4.0 International license.

Supplementary Figure S23. Correlation between glass former fragility (m-index) and DNA integrity in samples dried using the hotplate and humidified chamber methods under constant humidity and constant temperature conditions.
DNA integrity was assessed using the DNA Integrity Number (DIN). A-C show correlations for hotplate-dried samples at constant humidity (10%, 20%, and 50% RH), while D,E show correlations for humidified chamber-dried samples at constant humidity (10% and 20% RH; 50% RH not included as no fragility value was obtained). F-I show correlations for hotplate-dried samples at constant temperature (30 °C, 37 °C, and 44 °C), and I-K show correlations for humidified chamber-dried samples at constant temperature (30 °C, 37 °C, and 44 °C). Correlation coefficients (r) and significance values (p) were calculated using Pearson correlation for normally distributed data and Spearman correlation for non-normally distributed data. Each data point represents an individual replicate. Dashed lines indicate 95% confidence interval (CI).

bioRxiv preprint doi: https://doi.org/10.64898/2026.01.16.700019; this version posted January 17, 2026. The copyright holder for this preprint (which was not certified by peer review) is the author/funder, who has granted bioRxiv a license to display the preprint in perpetuity. It is made available under aCC-BY 4.0 International license.

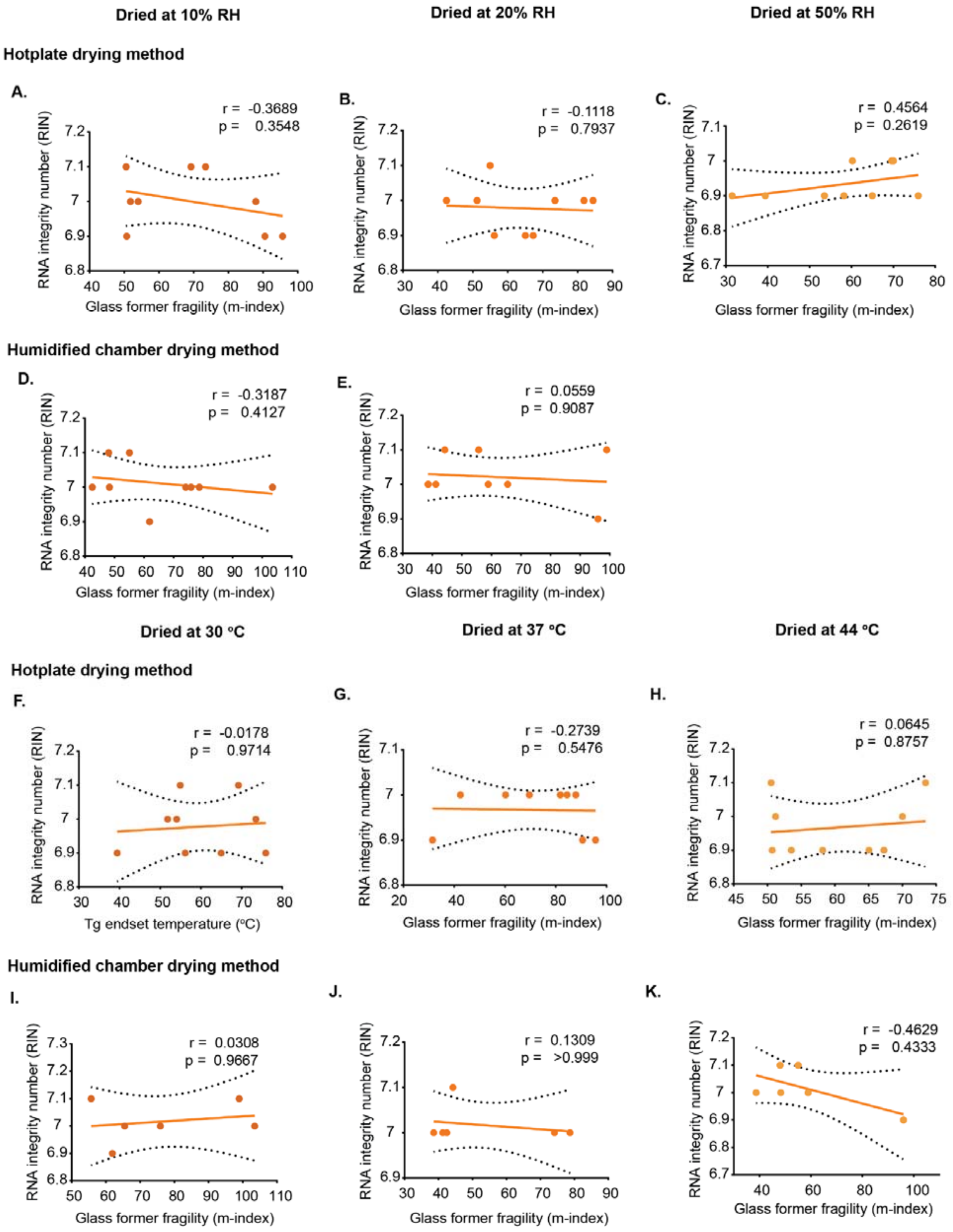

bioRxiv preprint doi: https://doi.org/10.64898/2026.01.16.700019; this version posted January 17, 2026. The copyright holder for this preprint (which was not certified by peer review) is the author/funder, who has granted bioRxiv a license to display the preprint in perpetuity. It is made available under aCC-BY 4.0 International license.

Supplementary Figure S24. Correlation between glass former fragility (m-index) and RNA integrity in samples dried using the hotplate and humidified chamber methods under constant humidity and constant temperature conditions.

RNA integrity was assessed using the RNA Integrity Number (RIN). A-C show correlations for hotplate-dried samples at constant humidity (10%, 20%, and 50% RH), while D,E show correlations for humidified chamber-dried samples at constant humidity (10% and 20% RH; 50% RH not included as no fragility value was obtained). F-I show correlations for hotplate-dried samples at constant temperature (30 °C, 37 °C, and 44 °C), and I-K show correlations for humidified chamber-dried samples at constant temperature (30 °C, 37 °C, and 44 °C). Correlation coefficients (r) and significance values (p) were calculated using Pearson correlation for normally distributed data and Spearman correlation for non-normally distributed data. Each data point represents an individual replicate. Dashed lines indicate 95% confidence interval (CI).

bioRxiv preprint doi: https://doi.org/10.64898/2026.01.16.700019; this version posted January 17, 2026. The copyright holder for this preprint (which was not certified by peer review) is the author/funder, who has granted bioRxiv a license to display the preprint in perpetuity. It is made available under aCC-BY 4.0 International license.

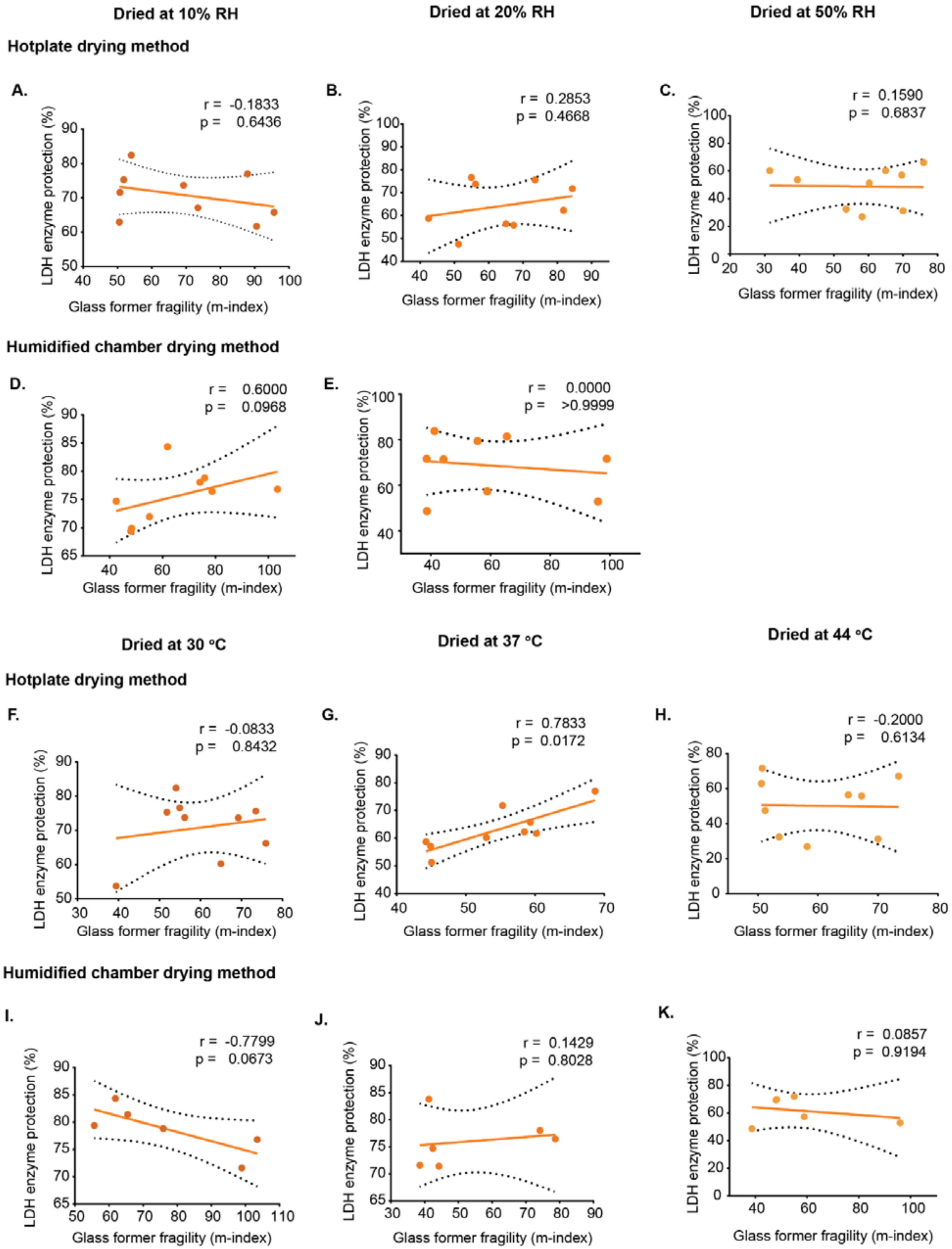

bioRxiv preprint doi: https://doi.org/10.64898/2026.01.16.700019; this version posted January 17, 2026. The copyright holder for this preprint (which was not certified by peer review) is the author/funder, who has granted bioRxiv a license to display the preprint in perpetuity. It is made available under aCC-BY 4.0 International license.

Supplementary Figure S25. Correlation between glass former fragility (m-index) and LDH activity in samples dried using the hotplate and humidified chamber methods under constant humidity and constant temperature conditions.

Lactate dehydrogenase (LDH) activity was expressed as the percentage of activity retained relative to the undried control. A-C show correlations for hotplate-dried samples at constant humidity (10%, 20%, and 50% RH), while D,E show correlations for humidified chamber-dried samples at constant humidity (10% and 20% RH; 50% RH not included as no fragility value was obtained). F-H show correlations for hotplate-dried samples at constant temperature (30 °C, 37 °C, and 44 °C), and I-K show correlations for humidified chamber-dried samples at constant temperature (30 °C, 37 °C, and 44 °C). Correlation coefficients (r) and significance values (p) were calculated using Pearson correlation for normally distributed data and Spearman correlation for non-normally distributed data. Each data point represents an individual replicate. Dashed lines indicate 95% confidence interval (CI).

bioRxiv preprint doi: https://doi.org/10.64898/2026.01.16.700019; this version posted January 17, 2026. The copyright holder for this preprint (which was not certified by peer review) is the author/funder, who has granted bioRxiv a license to display the preprint in perpetuity. It is made available under aCC-BY 4.0 International license.

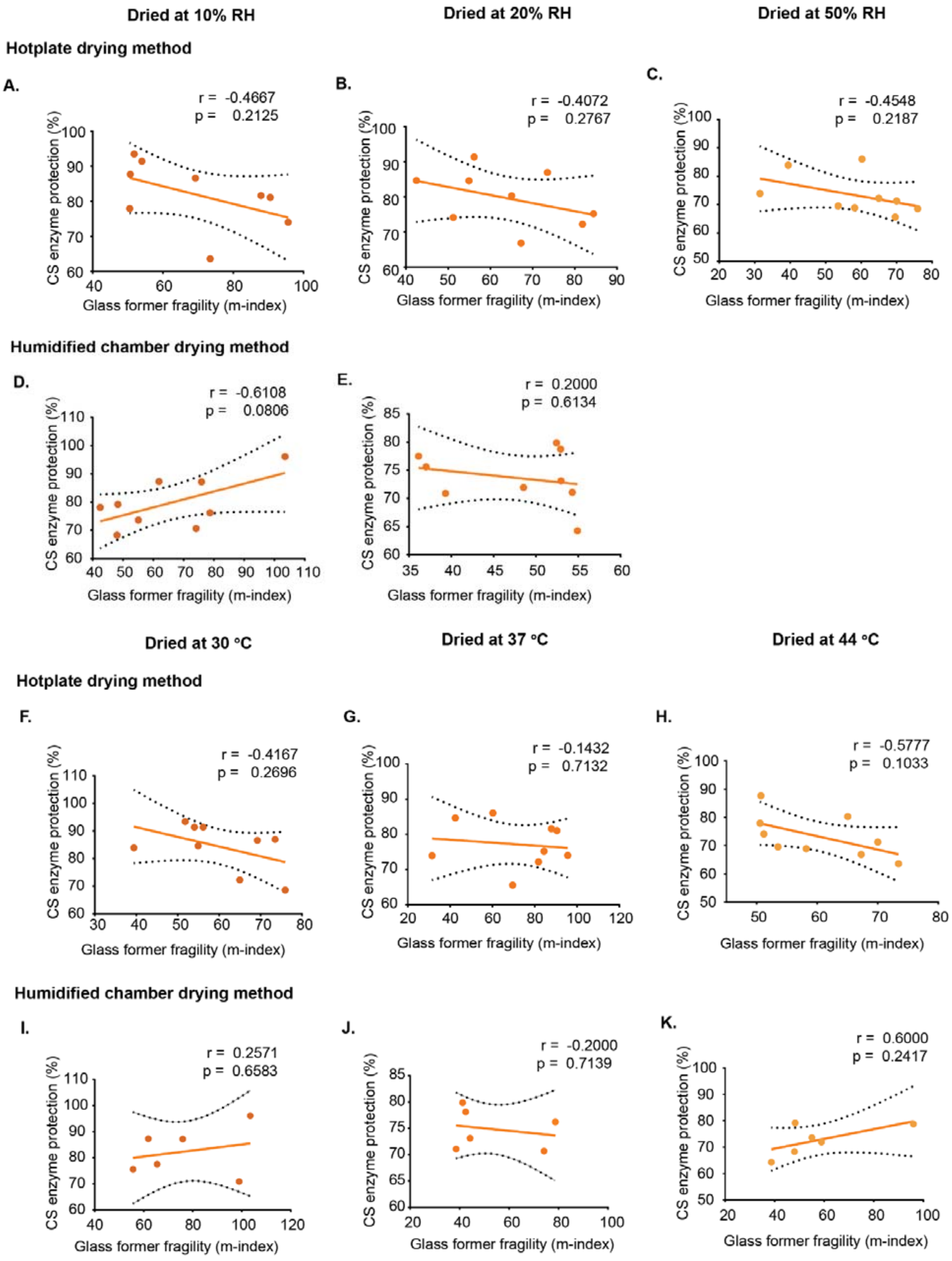

bioRxiv preprint doi: https://doi.org/10.64898/2026.01.16.700019; this version posted January 17, 2026. The copyright holder for this preprint (which was not certified by peer review) is the author/funder, who has granted bioRxiv a license to display the preprint in perpetuity. It is made available under aCC-BY 4.0 International license.

Supplementary Figure S26. Correlation between glass former fragility (m-index) and CS activity in samples dried using the hotplate and humidified chamber methods under constant humidity and constant temperature conditions.

A-C show correlations for hotplate-dried samples at constant humidity (10%, 20%, and 50% RH), while D,E show correlations for humidified chamber-dried samples at constant humidity (10% and 20% RH; 50% RH not included as no fragility value was obtained). F-H show correlations for hotplate-dried samples at constant temperature (30 °C, 37 °C, and 44 °C), and I-K show correlations for humidified chamber-dried samples at constant temperature (30 °C, 37 °C, and 44 °C). Correlation coefficients (r) and significance values (p) were calculated using Pearson correlation for normally distributed data and Spearman correlation for non-normally distributed data. Each data point represents an individual replicate. Dashed lines indicate 95% confidence interval (CI).

bioRxiv preprint doi: https://doi.org/10.64898/2026.01.16.700019; this version posted January 17, 2026. The copyright holder for this preprint (which was not certified by peer review) is the author/funder, who has granted bioRxiv a license to display the preprint in perpetuity. It is made available under aCC-BY 4.0 International license.

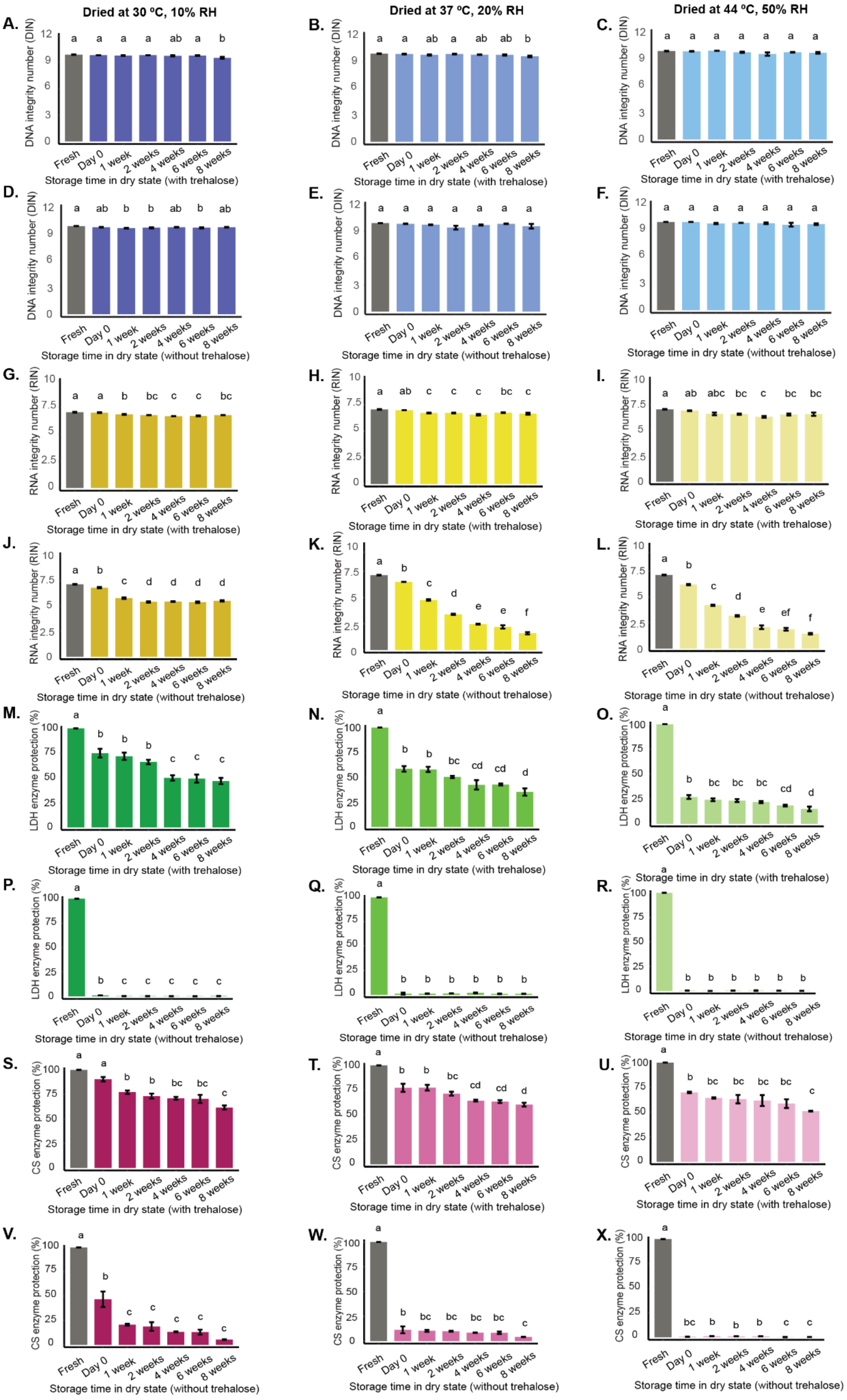

bioRxiv preprint doi: https://doi.org/10.64898/2026.01.16.700019; this version posted January 17, 2026. The copyright holder for this preprint (which was not certified by peer review) is the author/funder, who has granted bioRxiv a license to display the preprint in perpetuity. It is made available under aCC-BY 4.0 International license.

Supplementary Figure 27. Molecular stability during storage under different drying conditions with and without trehalose.

Samples of DNA (A–F), RNA (G–L), lactate dehydrogenase (LDH; M-R), and citrate synthase (CS; S-X) were dried using the hotplate method under three conditions: 30□°C at 10% RH (A, D, G, J, M, P, S, V), 37□°C at 20% RH (B, E, H, K, N, Q, T, W), and 44□°C at 50% RH (C, F, I, L, O, R, U, X). For each molecule, samples with trehalose are shown in the first three panels of each group (A-C, G-I, M-O, S-U), and samples without trehalose are shown in the next three panels (D-F, J-L, P-R, V-X). Samples were stored at room temperature in a 10% RH LiCl jar, and molecular stability was evaluated at Day 0, Week 1, Week 2, Week 4, and Week 8. Nucleic acid integrity and enzyme activity were assessed at each time point. Data represent mean ± SE from three independent experiments. Statistical comparisons were performed using one-way ANOVA followed by Tukey's post hoc test (α = 0.05); different letters indicate statistically significant differences among drying conditions within each panel.

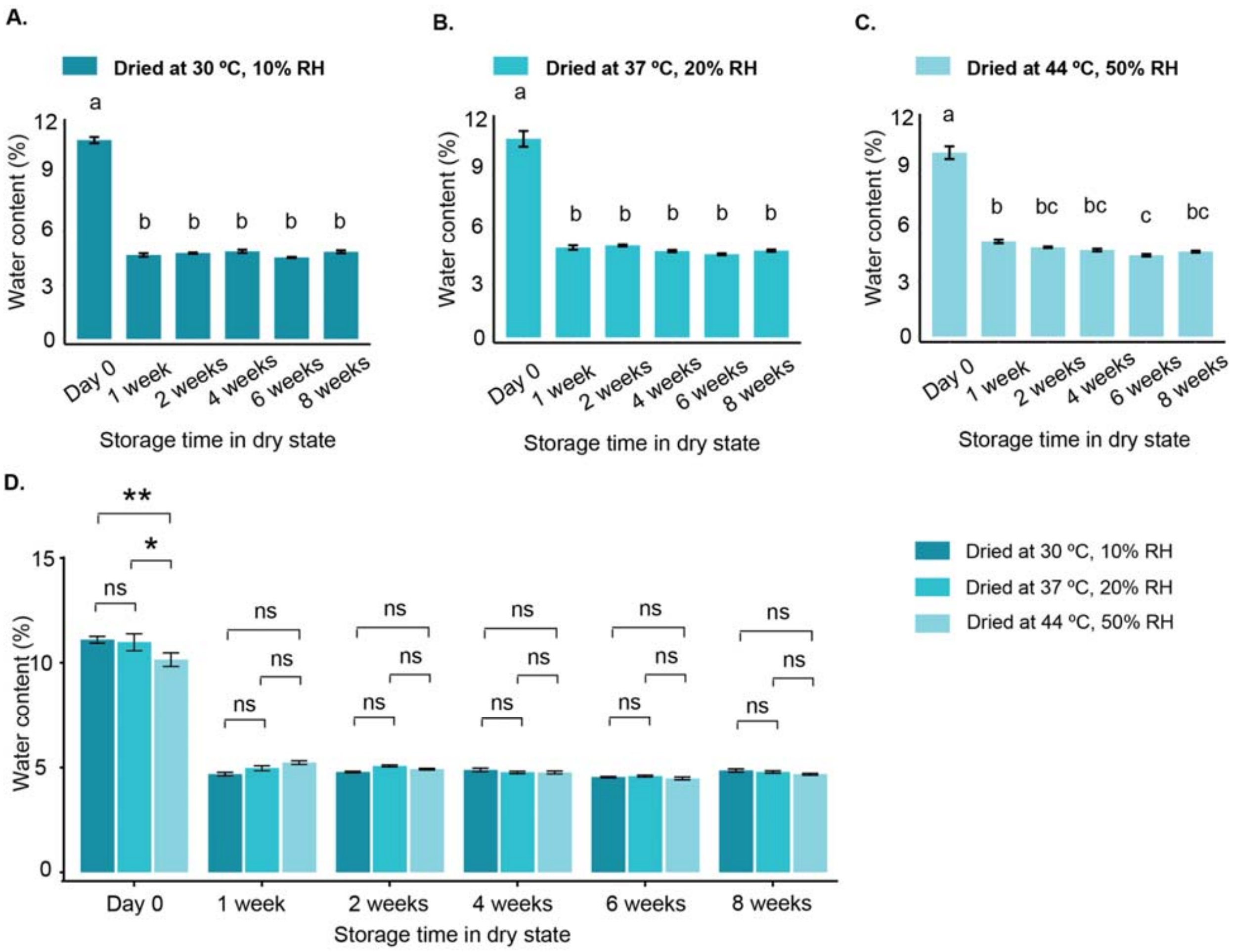

Supplementary Figure 28. Water content of dried samples during storage under different drying conditions.

Water content of samples dried under three conditions (A-C): 30 °C, 10% humidity (A); 37 °C, 20% humidity (B); and 44 °C, 50% humidity (C). Direct comparison of water content across the three drying conditions is shown in (D). Data are presented as mean ± SE. In panels A-C, different letters indicate statistical significance determined by one-way ANOVA followed by

bioRxiv preprint doi: https://doi.org/10.64898/2026.01.16.700019; this version posted January 17, 2026. The copyright holder for this preprint (which was not certified by peer review) is the author/funder, who has granted bioRxiv a license to display the preprint in perpetuity. It is made available under aCC-BY 4.0 International license.

Tukey's test. In panel D, statistical analysis was performed using two-way ANOVA with multiple comparisons.

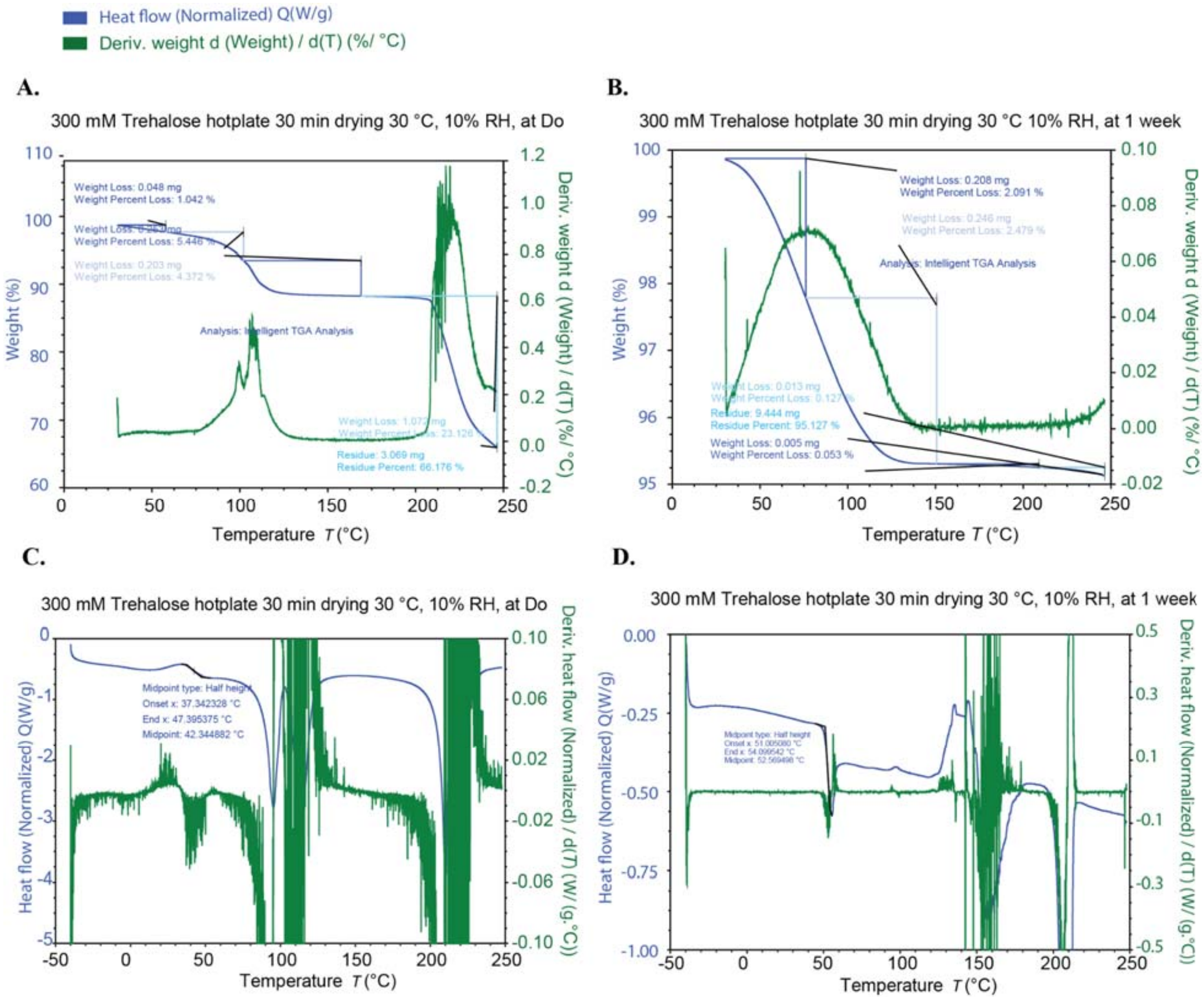

Supplementary Figure 29. Thermogravimetric analysis (TGA) and differential scanning calorimetry (DSC) thermographs of samples dried at 30 °C, 10% humidity.
A and B show TGA thermographs immediately after drying (A) and after 1 week of storage (B). C and D show DSC thermographs immediately after drying (C) and after 1 week of storage (D).

bioRxiv preprint doi: https://doi.org/10.64898/2026.01.16.700019; this version posted January 17, 2026. The copyright holder for this preprint (which was not certified by peer review) is the author/funder, who has granted bioRxiv a license to display the preprint in perpetuity. It is made available under aCC-BY 4.0 International license.

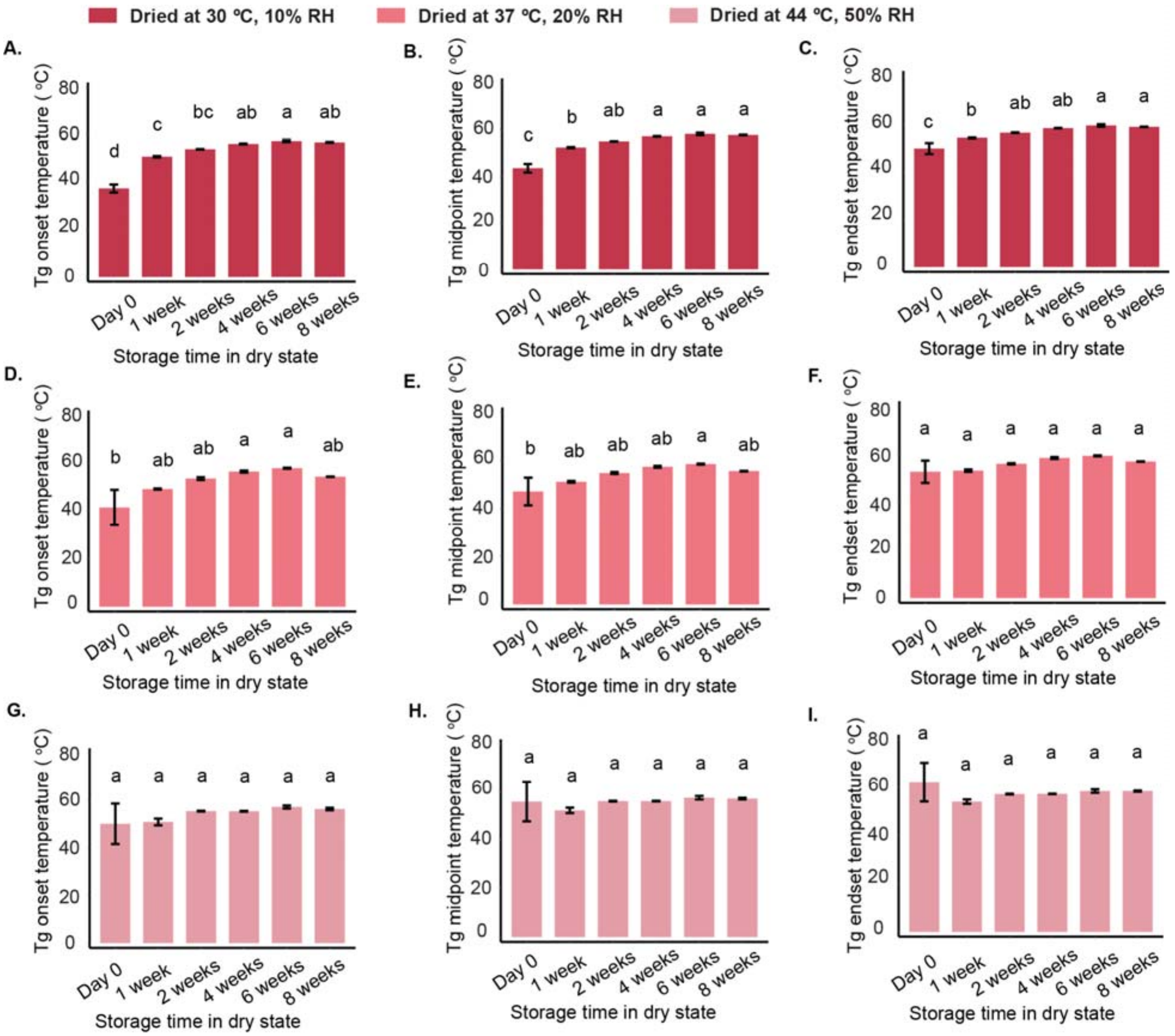

Supplementary Figure 30. Glass transition temperature (Tg) of dried samples during storage under different drying conditions.

Tg onset (A,D,G), midpoint (B,E,H), and endset (C,F,I) values of samples dried under three conditions are shown: 30 °C, 10% humidity (A-C); 37 °C, 20% humidity (D-F); and 44 °C, 50% humidity (G-I). Data are presented as mean ± SE. In each set of panels, different letters indicate statistical significance determined by one-way ANOVA followed by Tukey's test.

bioRxiv preprint doi: https://doi.org/10.64898/2026.01.16.700019; this version posted January 17, 2026. The copyright holder for this preprint (which was not certified by peer review) is the author/funder, who has granted bioRxiv a license to display the preprint in perpetuity. It is made available under aCC-BY 4.0 International license.

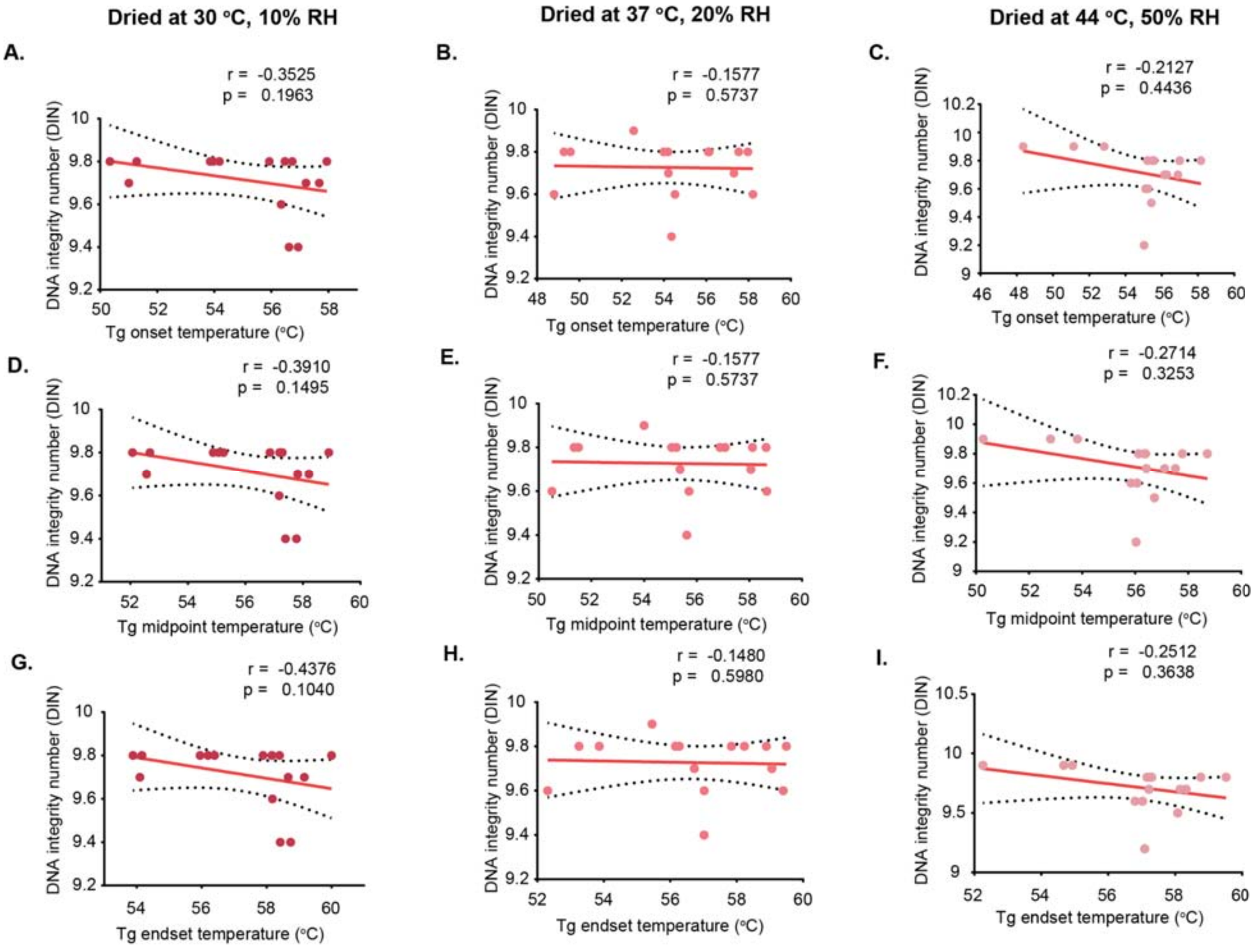

Supplementary Figure 31. Correlation between DNA integrity and glass transition temperature (Tg) under different drying conditions during storage.

Correlations between DNA integrity, assessed using the DNA Integrity Number (DIN), and Tg values are shown for three drying conditions: 30 °C, 10% humidity (A,D,G); 37 °C, 20% humidity (B,E,H); and 44 °C, 50% humidity (C,F,I). A-C show correlations with Tg onset, D-F show correlations with Tg midpoint, and G-I show correlations with Tg endset. Correlation coefficients (r) and significance values (p) were calculated using Pearson correlation for normally distributed data and Spearman correlation for non-normally distributed data. Each data point represents an individual replicate. Dashed lines indicate 95% confidence interval (CI).

bioRxiv preprint doi: https://doi.org/10.64898/2026.01.16.700019; this version posted January 17, 2026. The copyright holder for this preprint (which was not certified by peer review) is the author/funder, who has granted bioRxiv a license to display the preprint in perpetuity. It is made available under aCC-BY 4.0 International license.

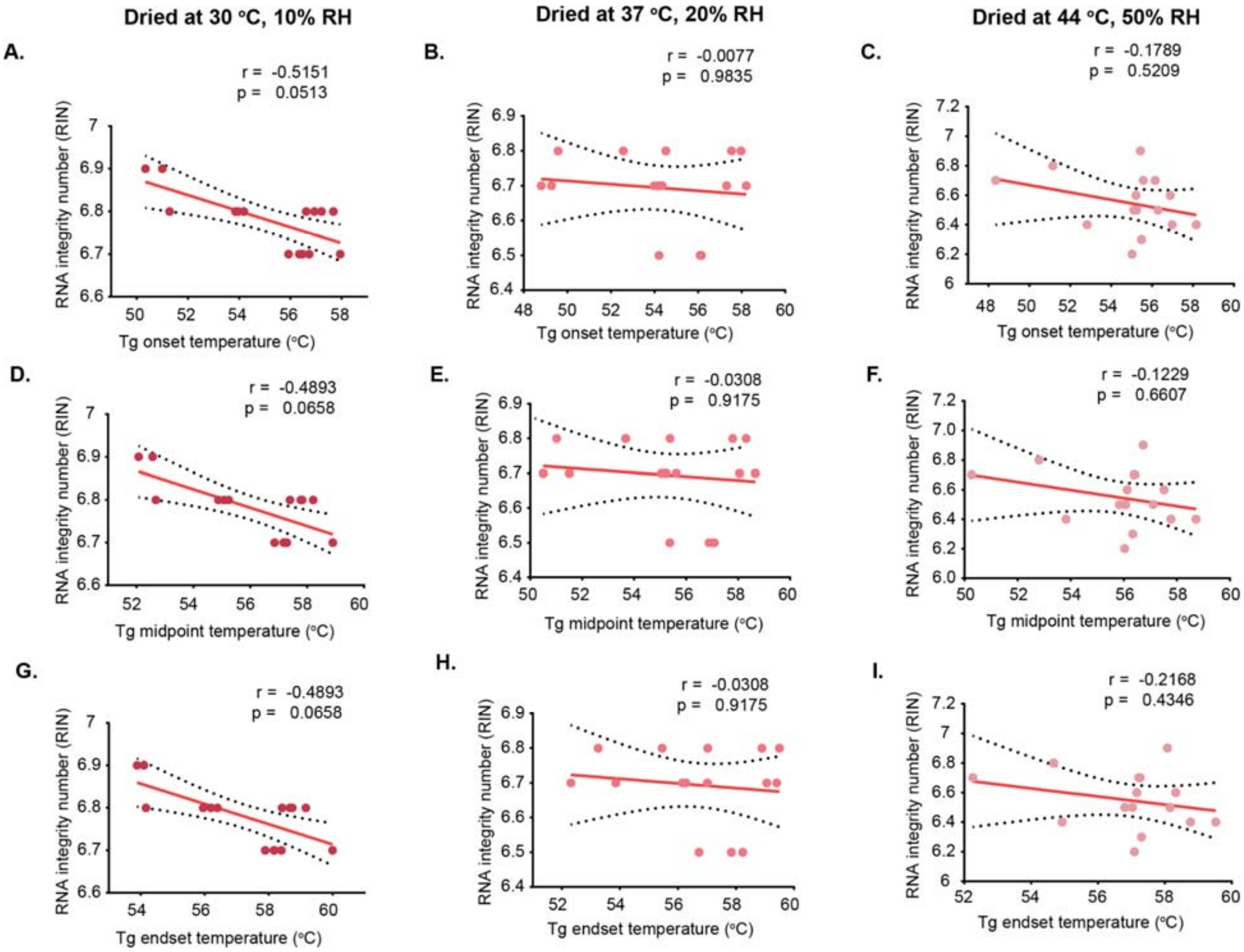

Supplementary Figure 32. Correlation between RNA integrity and glass transition temperature (Tg) under different drying conditions during storage.

Correlations between RNA integrity, assessed using the RNA Integrity Number (RIN), and Tg values are shown for three drying conditions: 30 °C, 10% humidity (A,D,G); 37 °C, 20% humidity (B,E,H); and 44 °C, 50% humidity (C,F,I). A-C show correlations with Tg onset, D-F show correlations with Tg midpoint, and G-I show correlations with Tg endset. Correlation coefficients (r) and significance values (p) were calculated using Pearson correlation for normally distributed data and Spearman correlation for non-normally distributed data. Each data point represents an individual replicate. Dashed lines indicate 95% confidence interval (CI).

bioRxiv preprint doi: https://doi.org/10.64898/2026.01.16.700019; this version posted January 17, 2026. The copyright holder for this preprint (which was not certified by peer review) is the author/funder, who has granted bioRxiv a license to display the preprint in perpetuity. It is made available under aCC-BY 4.0 International license.

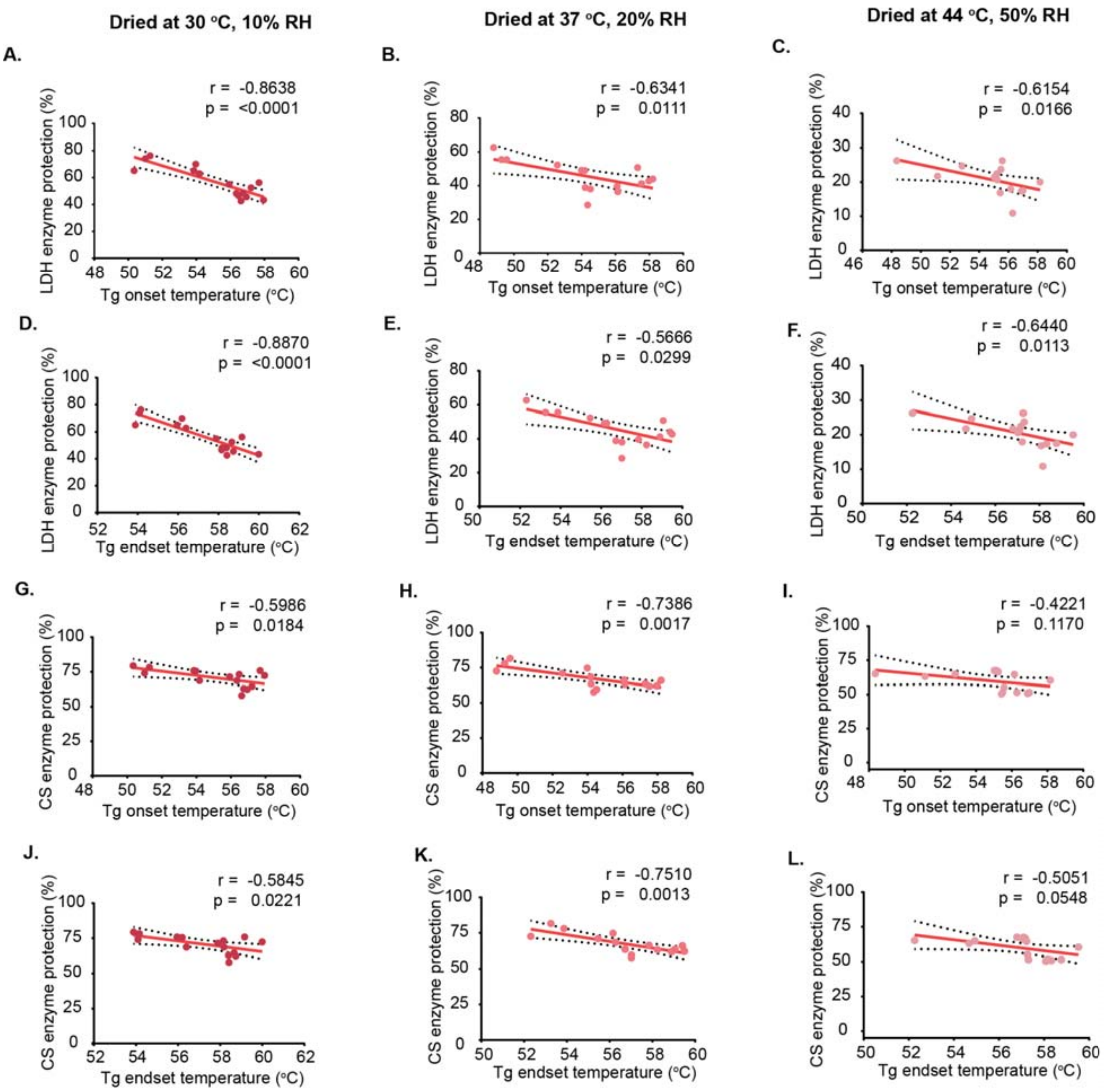

Supplementary Figure 33. Correlation between glass transition onset and endset temperatures (Tg) and enzyme activity (LDH and CS) under different drying conditions over storage time.

Samples were dried under three conditions: 30 °C, 10% humidity (A, D, G, J); 37 °C, 20% humidity (B, E, H, K); and 44 °C, 50% humidity (C, F, I, L). Correlations between Tg and enzyme activity are shown for lactate dehydrogenase (LDH; A-F) and citrate synthase (CS; G-L). Pearson correlation was used for normally distributed data, and Spearman correlation was used for non-normally distributed data. Each data point represents an individual replicate. Dashed lines indicate 95% confidence interval (CI).

bioRxiv preprint doi: https://doi.org/10.64898/2026.01.16.700019; this version posted January 17, 2026. The copyright holder for this preprint (which was not certified by peer review) is the author/funder, who has granted bioRxiv a license to display the preprint in perpetuity. It is made available under aCC-BY 4.0 International license.

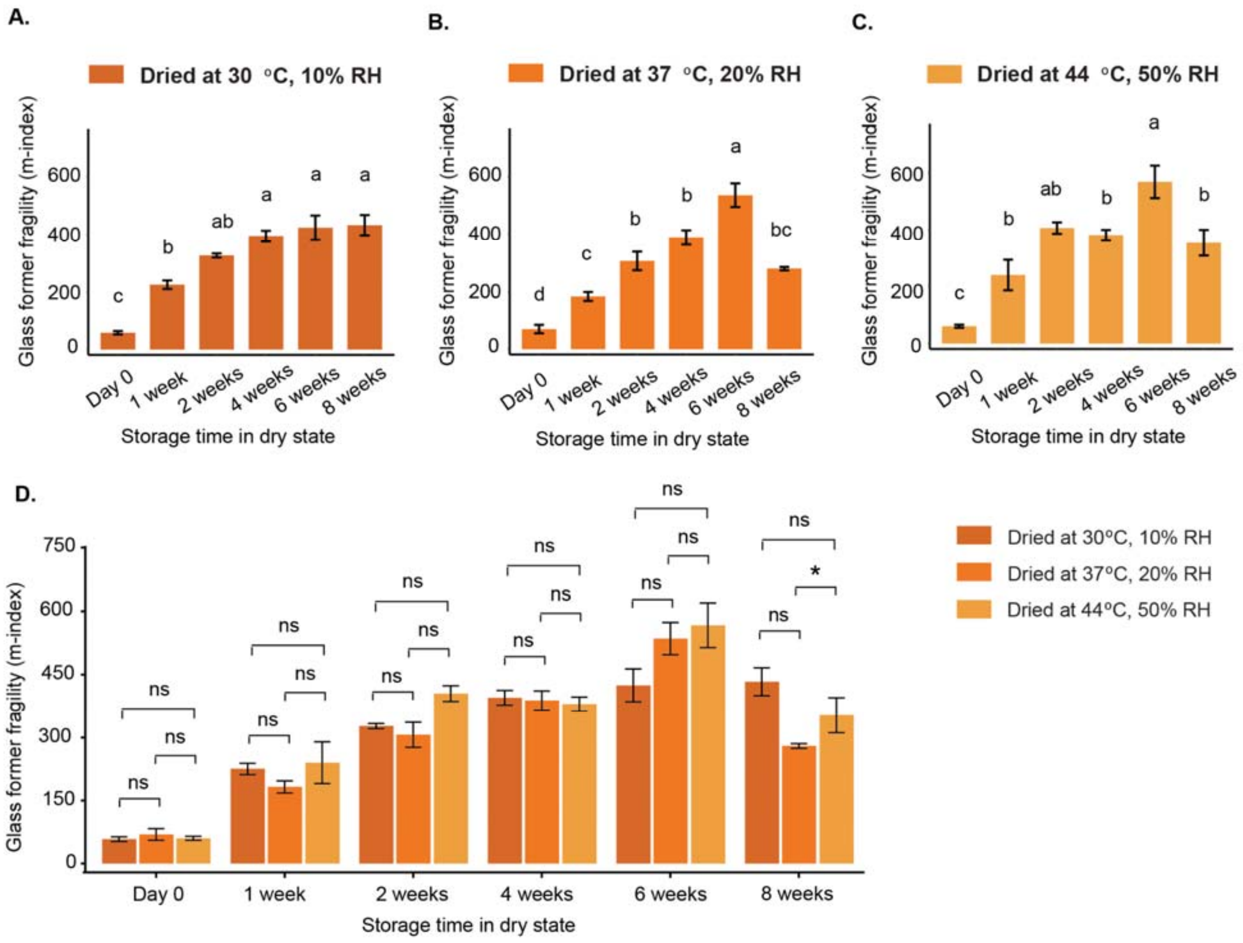

Supplementary Figure 34. Glass former fragility (m-index) of dried samples during storage under different drying conditions.

Fragility values of samples dried under three conditions (A-C): 30□°C, 10% humidity (A); 37□°C, 20% humidity (B); and 44□°C, 50% humidity (C). Direct comparison of fragility across the three drying conditions is shown in (D). Data are presented as mean ± SE. In panels A-C, different letters indicate statistical significance determined by one-way ANOVA followed by Tukey's test. In panel D, statistical analysis was performed using two-way ANOVA with multiple comparisons.

bioRxiv preprint doi: https://doi.org/10.64898/2026.01.16.700019; this version posted January 17, 2026. The copyright holder for this preprint (which was not certified by peer review) is the author/funder, who has granted bioRxiv a license to display the preprint in perpetuity. It is made available under aCC-BY 4.0 International license.

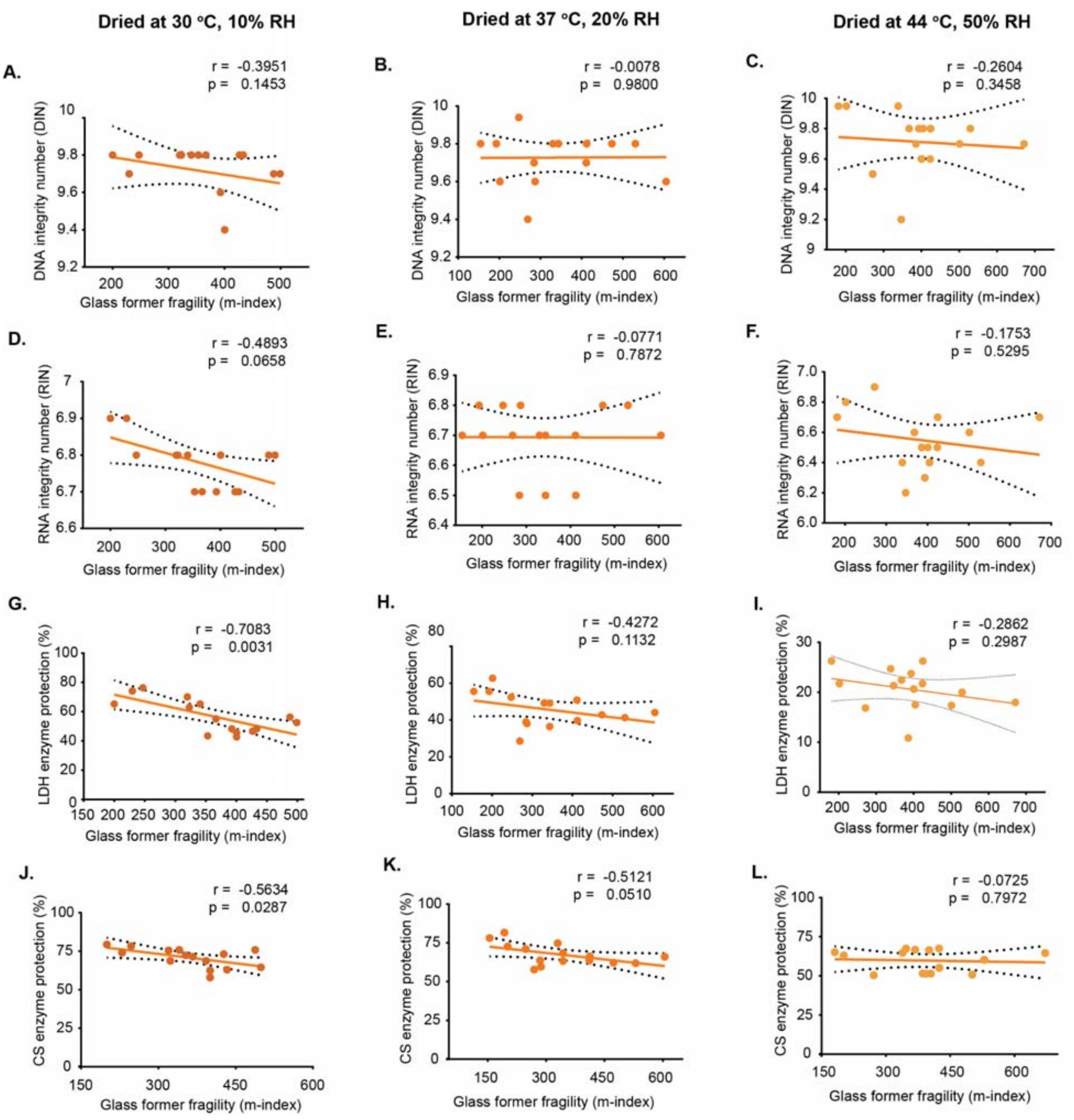

Supplementary Figure 35. Correlation between glass former fragility (m-index) and nucleic acid integrity or protein activity under different drying conditions during storage. Samples were dried under three conditions: 30□°C, 10% humidity (A, D, G, J); 37□°C, 20% humidity (B, E, H, K); and 44□°C, 50% humidity (C, F, I, L). Correlations are shown for DNA integrity (A-C), RNA integrity (D-F), lactate dehydrogenase (LDH) activity (G-I), and citrate synthase (CS) activity (J-L). Pearson correlation was used for normally distributed data, and Spearman correlation was used for non-normally distributed data. Each data point represents an individual replicate. Dashed lines indicate 95% confidence interval (CI).

bioRxiv preprint doi: https://doi.org/10.64898/2026.01.16.700019; this version posted January 17, 2026. The copyright holder for this preprint (which was not certified by peer review) is the author/funder, who has granted bioRxiv a license to display the preprint in perpetuity. It is made available under aCC-BY 4.0 International license.

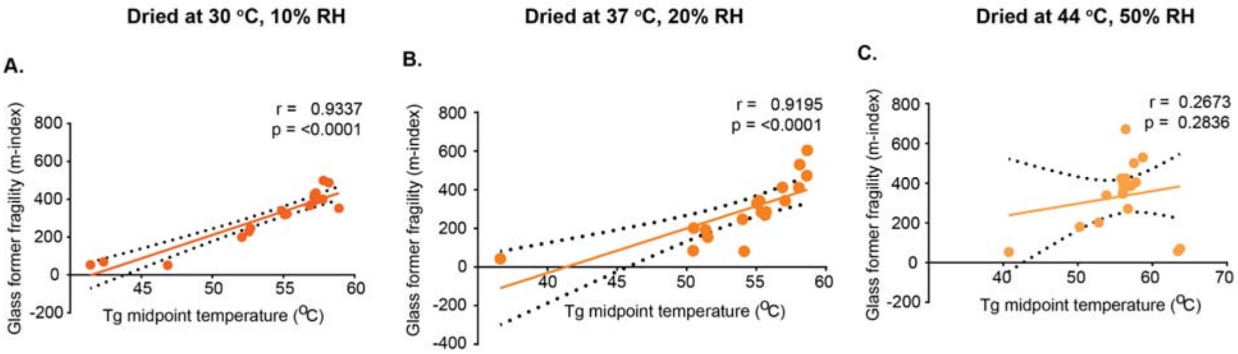

Supplementary Figure S36. Correlation between glass transition (Tg) midpoint temperature and glass former fragility (m-index) under different drying conditions during storage.

Samples were dried under three conditions: 30 °C, 10% humidity (A); 37 °C, 20% humidity (B); and 44 °C, 50% humidity (C). Correlation coefficients (r) and significance values (p) were calculated using Pearson correlation for normally distributed data and Spearman correlation for non-normally distributed data. Each data point represents an individual replicate. Dashed lines indicate 95% confidence interval (CI).
